# Molecular Evolution and Expression Divergence of *HMT* Gene Family in Plants

**DOI:** 10.3390/ijms19041248

**Published:** 2018-04-20

**Authors:** Man Zhao, Peng Chen, Wenyi Wang, Fengjie Yuan, Danhua Zhu, Zhao Wang, Xiangxian Ying

**Affiliations:** 1 College of Biotechnology and Bioengineering, Zhejiang University of Technology, Hangzhou 310014, China; chenpengoffice@163.com (P.C.); hzwywang@163.com (W.W.); hzwangzhao@163.com (Z.W.); 2 Institute of Crop Science, Zhejiang Academy of Agricultural Sciences, Hangzhou 310014, China; fjyuanhz@126.com (F.Y.); danhua163@hotmail.com (D.Z.)

**Keywords:** homocysteine methyltransferase, *HMT* genes, evolution, gene expression, methyl donors

## Abstract

Homocysteine methyltransferase (HMT) converts homocysteine to methionine using *S*-methylmethionine (SMM) or *S*-adenosylmethionine (SAM) as methyl donors in organisms, playing an important role in supplying methionine for the growth and the development of plants. To better understand the functions of the *HMT* genes in plants, we conducted a wide evolution and expression analysis of these genes. Reconstruction of the phylogenetic relationship showed that the *HMT* gene family was divided into Class 1 and Class 2. In Class 1, *HMT*s were only found in seed plants, while Class 2 presented in all land plants, which hinted that the *HMT* genes might have diverged in seed plants. The analysis of gene structures and selection pressures showed that they were relatively conserved during evolution. However, type I functional divergence had been detected in the *HMT*s. Furthermore, the expression profiles of *HMT*s showed their distinct expression patterns in different tissues, in which some *HMT*s were widely expressed in various organs, whereas the others were highly expressed in some specific organs, such as seeds or leaves. Therefore, according to our results in the evolution, functional divergence, and expression, the *HMT* genes might have diverged during evolution. Further analysis in the expression patterns of *AthHMT*s with their methyl donors suggested that the diverged *HMT*s might be related to supply methionine for the development of plant seeds.

## 1. Introduction

Methionine (Met) is an essential amino acid in humans that is obtained through diets. Cereal and legume crops are the main food sources for the humans; however, they contain limited essential amino acids [1,2]. Met plays important functions in the synthesis of proteins and in the initiation of mRNA translation. It also indirectly regulates various metabolic processes through its main catabolic products *S*-adenosylmethionine (SAM, AdoMet) [1,2,3]. SAM is not only the methyl donor to many compounds, but it is also the precursor of many important metabolites, such as the polyamines spermidine, nicotianamine, phytosiderophores, ethylene, and so on [2,3]. The nutritional and functional roles of Met suggest that it is necessary to reveal the biosynthesis and the accumulation of Met in plants.

The biosynthesis of Met is involved in two pathways in plants. Firstly, Met can be synthesized de novo through the aspartate family pathway, which is mainly catalyzed by cystathionine γ-synthase (CGS) and methionine synthase (MS). Secondly, the *S*-methylmethionine (SMM) cycle to synthesize Met has also been proposed in plants. In this cycle, SMM and homocysteine (Hcy) are catalyzed by homocysteine methyltransferase (HMT) into two molecules of Met, while SMM is formed by methionine methyltransferase (MMT) through catalyzing the reaction of Met and SAM [4,5,6,7]. Nevertheless, the HMT and MMT perform functions with their own spatial and temporal specificity. Isotope-labeling experiments indicated that SMM is mainly synthesized by MMT in vegetative tissues, such as leaves, and is then transported into the developing flowers and seeds through the phloems, in which SMM is reconverted back into Met by the catalysis of HMT [4,6]. Therefore, to maintain the SMM cycle, MMT activity is high in leaves and low in seeds and flowers, while the HMT activity is opposite [4,6,7,8,9]. Further, the SMM cycle has been suggested to mainly contribute to the accumulation of Met in the plant seeds. 

Although the SMM cycle is only presented in plants, *HMT*s have been widely found in bacteria, fungi, animals, and plants. In animals, owing to their important roles in health and nutrition, *BHMT* genes have been widely studied in different species, and their evolutionary history has also been investigated [10,11]. In mammals, studies have found that *BHMT* diverged into *BHMT* (Betaine-homocysteine *S*-methylmethionine) and *BHMT2*, which used betaine and SAM as the methyl donors, respectively [11,12]. Furthermore, the two copies experienced different functional divergence and selection pressures during evolution. In plants, *HMT* genes have been identified in a limited number of species, such as *Arabidopsis thaliana*, *Medicago truncatula*, *Zea mays,* and so on, but they only were investigated in *Arabidopsis* in detail [5,8,13,14,15].

In *Arabidopsis*, there are three copies of *HMT*: *AtHMT1* (*AthHMT1* in this study), *AtHMT2* (*AthHMT2* in this study) and *AtHMT3* (*AthHMT3* in this study). Studies have revealed that the expression levels of *AtHMT*s are varied in different tissues. The expression of *AtHMT1* and *AtHMT3* is dominant in the developing seeds, especially in the maturing stage, while *AtHMT2* does not show special fluctuation during the whole life of *Arabidopsis* [4,7,15]. Nevertheless, their functions, converting Hcy into methionine using AdoMet or SMM as the methyl donor, are conserved. Generally, AdoMet contains the *R*,*S-* and *S*,*S-* forms, and *S*,*S-* is the natural active form. In physiological conditions, the *S*,*S*-AdoMet is labile and it is easy to convert into *R*,*S*-AdoMet suffering chemical damage reactions [16,17]. In *Arabidopsis*, the methyl donors of AtHMTs differ: the donors for AtHMT1 are SMM and *R*,*S*-AdoMet, while the donors for AtHMT2 and AtHMT3 are *R*,*S*-AdoMet and SMM, respectively. Additionally, Bradbury’s research also suggested that the ancestral function of HMT was to repair *R*,*S*-AdoMet, while the SMM first occurred in the world at the time of the advent of plants [17]. Nevertheless, the evolutionary process of *HMT* in plants has not been investigated, and even less is known about whether the functions of HMTs have diverged within plants.

Based on the important roles of *HMT*s in plants, in this study, we comprehensively analyzed the evolutionary history and structures of *HMT* genes, examined their selection pressures, and predicted their functional divergence and corresponding diverged sites. Furthermore, we analyzed their expression and their promoter regions. Taken together, the evolutionary history and the functional divergence of the *HMT* gene family in plants were widely investigated. 

## 2. Results

### 2.1. Diverged HMT Genes in Seed Plants

To reconstruct the evolutionary relationship of *HMT* genes in plants, the *HMT* genes were widely identified in plants. In all, 75 *HMT* sequences were analyzed in representative 29 species (Table S1 in Additional file 1). The species that we chose represented the width of the plants lineages (Tables S1 and S2 in Additional file 1). The copies of *HMT* varied from 1 to 4 in each species, and the lengths of *HMT*s ranged from 840 to 1353 bp (Tables S1–S3 in Additional file 1). 

**Figure 1:**
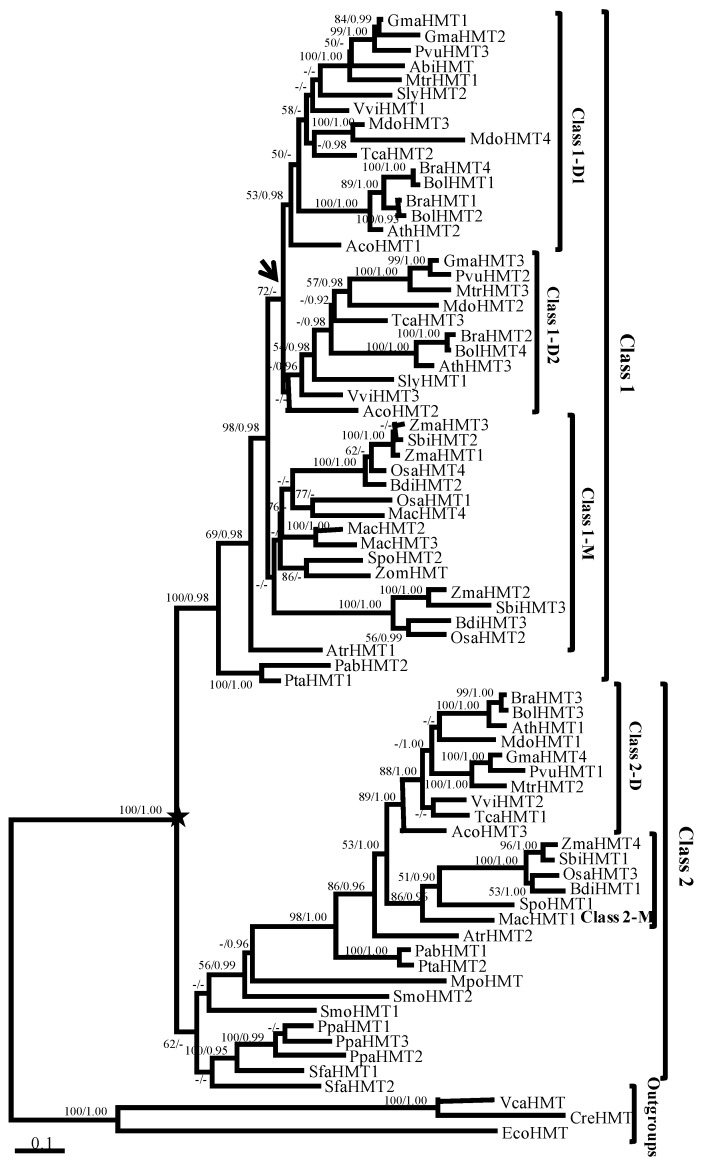
Phylogenetic tree of the homocysteine methyltransferase (*HMT)* gene family in plants. The tree was constructed with ML and Bayesian methods based on the amino acid (aa) sequences with EcoHMT, CreHMT, and VcaHMT as outgroups. Support values (>50% of ML) and posterior probabilities (>0.9 of Bayesian) for this tree are shown on respective branches. The black star indicates the divergence of Class 1 and Class 2, and the arrow indicates the divergence of Class 1-D1 and Class 1-D2. Gene names and identifiers are shown in Table S1 in Additional file 1.

The phylogenetic trees of the *HMT* genes were reconstructed using maximum likelihood (ML) and Bayesian methods. Given their similar topologies, the ML tree was displayed in Figure 1 because of its higher support values. Phylogenetically, the *HMT*s from *Escerichia coli* and two chlorophyta species that were located at the base of the phylogenetic tree were used as outgroups. The genes in land plants were divided into Class 1 and Class 2, with 100% support value (marked by a black star in Figure 1). In Class 1, the *HMT*s were found only in seed plants. Moreover, the genes in the eudicots diverged into two clades, Class 1-D1 and Class 1-D2 (marked by the black arrow in Figure 1), but the support value was relatively low, which suggested their ambiguous relationship. By contrast, Class 2 contained all land plant lineages, such as angiosperms, gymnosperms, and basal land plants (Figure 1 and Table S2 in Additional file 1), but the gene number in Class 2 (27 genes) was less than that in Class 1 (45 genes). Notably, the support values of the *HMT* genes in basal land plants were low (marked in red box in Figure 1), which hinted at their undefined phylogenetic relationship. Nevertheless, the *HMT* genes diverged into two classes in seed plants, which suggested that the functions of *HMT* genes might have diverged during evolution. 

### 2.2. Conserved Gene Structures of HMT Genes in Plants

The divergence of genes is partly shown in their structures. In our study, the intron-exon structures of *HMT*s were first analyzed. We compared the numbers and lengths of introns and exons in plants. Three *HMT* genes were not included because of their unavailable gene sequences (Table S3 in Additional file 1). Statistically, 70.67% (53/75 = 70.67%, Table S3 in Additional file 1) *HMT*s in plants were conserved containing seven exons and six introns. The intron-exon structures of the remaining *HMT* genes (22/75 = 29.33%) were diverged, in which 14 genes divergences were from intron loss or gain events of conserved *HMT*s (14/22 = 63.64%, intron losses and gains are marked in blue and red, respectively, in Table S3 in Additional file 1). Hence, the numbers of the introns and exons in the *HMT* genes were relatively conserved. Besides the numbers, the lengths of corresponding exons were basically consistent, while in introns the lengths were diverse.

**Figure 2:**
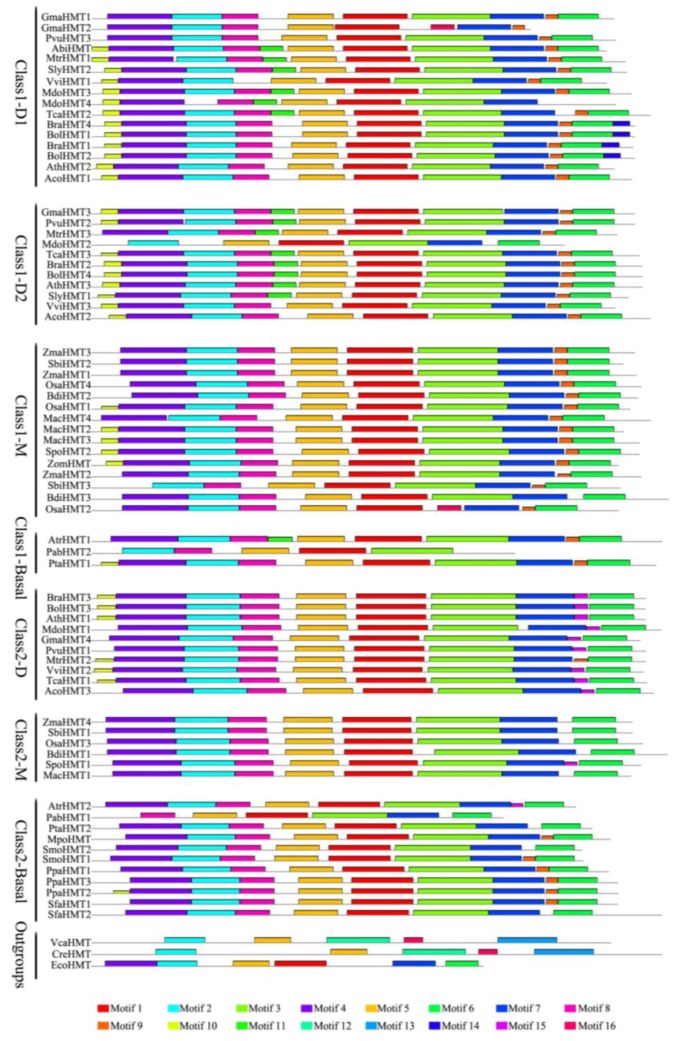
Conserved motifs of HMT proteins identified on the MEME analysis across plants. Each motif is represented by a colored box numbered on the bottom*.* The amino acid sequences of these motifs are presented in Figure S1 in Additional file 3. The black lines represent unique sequences. The scale bar indicates number of amino acids. Names to the left indicate the clades to which the sequences belong to according to Figure 1.

Additionally, the HMT proteins were also analyzed to survey the conserved protein motifs on Multiple Em for Motif Elicitation (MEME). Overall, 16 motifs were identified, in which the motifs from the motif 1 to motif 8 were conserved across almost all of the sequences, while some motifs ranging from the motif 9 to motif 16 only presented in some of the lineages (Figure 2, Figure S1 in Additional file 3). The conserved motifs, especially from motif 1 to motif 8, were also very important to the function of HMTs. For example, the residue Tyr^94^ in motif 1 and Thr^274^ in motif 2, which existed in all of the plants except for the two chlorophyta species, were critical for HMT enzyme activity (Figure 2, Figure S1 in Additional file 3, Additional file 2) [18]. Similarly, three important cysteine residues (Cys^350^, Cys^495^, Cys^496^ in alignment, Additional file 2), which formed a unique Zn^2+^-binding center for catalysis, were located in motif 3 and motif 6 (Figure 2, Figure S1 in Additional file 3, Additional file 2) [18]. Moreover, two important loop regions (α’5/ α’6 region and the missing loop α7/β7 are marked with black lines in Additional file 2) were found in motif 5 and motif 7 (Figure 2, Figure S1 in Additional file 3, Additional file 2) [18]. Additionally, the motifs pattern in each clade was basically similar in all of the plants, except for the outgroups, which indicated that the protein motifs of the HMT family were relatively conserved during evolution, especially in the important sites and proteins (Figure 2). 

### 2.3. Stringent Adaptive Evolution of HMT Gene Family

Selection pressure could also reflect the divergence of the gene family during evolution. In order to investigate the adaptive evolution of the *HMT* gene family during evolution, we estimated the *ω* values (*ω* = *dN*/*dS*), which are defined as the ratios to nonsynonymous and synonymous substitution. According to the results, M0, the null hypothesis, was compared with different alternative hypotheses: M2 (Class 1) and M2 (Class 2) (Table S4 in Additional file 1). No positive selection was detected in the two classes, and both of the *ω* values were 0.16, indicating that they were under strong purifying selection. However, adaptive evolution may only occur at a few time points and affect a few amino acids; thus, the branch-site models were executed in our study. By comparing model A null and model A [19], the significant positive selection was detected in the branches of Class 1 (*p* < 0.05) and Class 2 (*p* < 0.01). Only one site (position 97 in alignment) with a Bayes Empirical Bayes (BEB) of more than 0.95 was detected in Class 2, but no site was detected in Class 1. Taken into account, the *HMT* genes were well conserved, except for a slight divergence between Class 1 and Class 2 that occurred during evolution. 

### 2.4. Functional Divergence of HMT Proteins

To further study the divergence of HMT proteins in plants, we estimated the type I and type II functional divergences between clades using the software Diverge 3.0 (Xun Gu, Iowa State University, Ames, IA, USA). As for type I functional divergences, our results revealed that they were significant between Class 1 and Class 2 (θI = 0.13 ± 0.04, *p* = 0.0008), and Class 1-D and Class 2-D (θI = 0.20 ± 0.05, *p* = 1.3 × 10^−5^), but not in Class 1-D1 and Class 1-D2, and Class 1-M and Class 2-M (Table 1). Six diverged sites were detected between Class 1 and Class 2. When we matched them into the secondary structure of the HMT proteins, four of the six critical sites were found to be located in the α helixes or β sheets in the secondary structure of HMT proteins, the remaining two were located was in the loop regions (Table S5 in Additional file 1). Similarly, six of the 11 sites between Class 1-D and Class 2-D were found to be located in α helixes or β sheets, and the remaining five were located in the loops between α helixes or β sheets. As for type II functional divergence (FD II), no divergence was detected in any of the groups. Generally, only type I functional divergence was detected during evolution, which hinted that the divergence of the HMT family was not critical.

**Figure 3:**
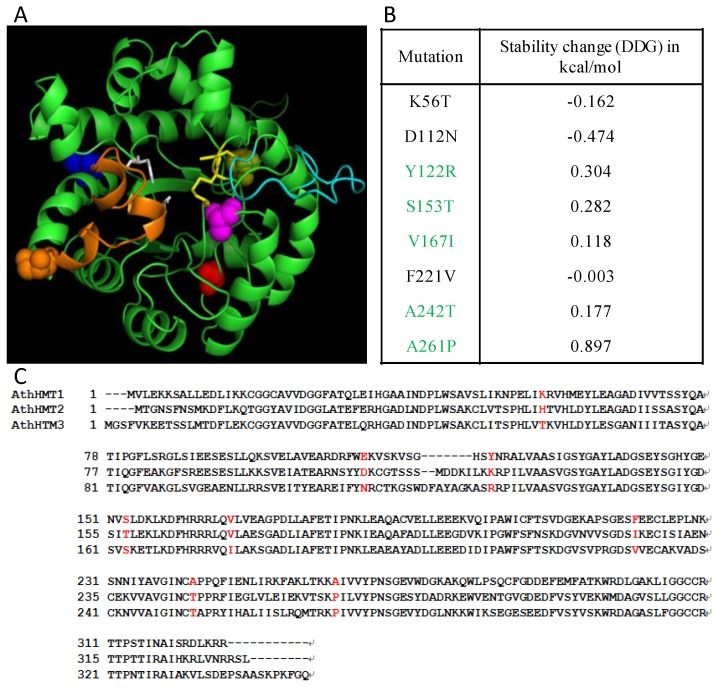
Potential structural changes in AthHMT1. (**A**) Overlap of the structural model of AthHMT1 in green, using PyMOL V2.1.1 (https://pymol.org). The different color spheres indicated the predicted key sites of 122 (yellow), 153 (orange), 167 (blue), 242 (magenta), and 261 (red) in AthHMT1; the yellow sticks denote the key sites for the enzyme activity, the white represents three Cys residues (241, 308, and 309 residues) composing a Zn^2+^-binding center, and the orange, and cyan sheets represent the important loops in the protein, which were reported in *E. coli* by Li et al [18]. (**B**) Stability calculations for mutations of AthHMTs performed by the Impact of Non-synonymous mutations on Protein Stability-Multi Dimension (INPS-MD), the ∆G of sites in green were above 0. (**C**) Consensus sequences for AthHMTs. The sites in red represent type I functional divergence. The cylinders and arrows represent α helixes and β sheets, respectively. The solid lines represent the random coils in the AthHMTs.

To detect the influence of the critical sites to the protein functions, we matched them to the three-dimensional (3D) protein structures and calculated their structure stability. The 3D-structural model of AthHMT1 was generated in I-TASSER, and the predicted sites were distributed through the whole protein (Figure 3). The structure stability of the mutations in AthHMT1 consensus sequences was predicted. The results revealed that five sites, Y to R in 122 (∆G = 0.304), S to T in 153 (∆G = 0.282), V to I in 167 (∆G = 0.118), A to T in 242 (∆G = 0.177), and A to P in 261 (∆G = 0.897), were unfavorable for structure stability, because their changes in ∆G were above 0 (Figure 3 and Table S5 in Additional file 1). When the five sites were located in the spatial structure of AthHMT1, we found four sites located in the loops between α helix and β sheet, except for site 167, which was located in α3 (Figure 3 and Table S5 in Additional file 1). Additionally, sites 153 and 242 (marked in orange and magenta spheres in Figure 3) were close to the catalysis center of HMT in one end of the triosephosphate isomerase (TIM) barrel, while sites 122 and 261 (marked in yellow and red spheres in Figure 3) were in the other end. Therefore, the four sites likely functioned to maintain the structure of the TIM barrel, and probably influenced the catalysis of the substrates.

### 2.5. Different Expression Patterns of HMTs

**Figure 4:**
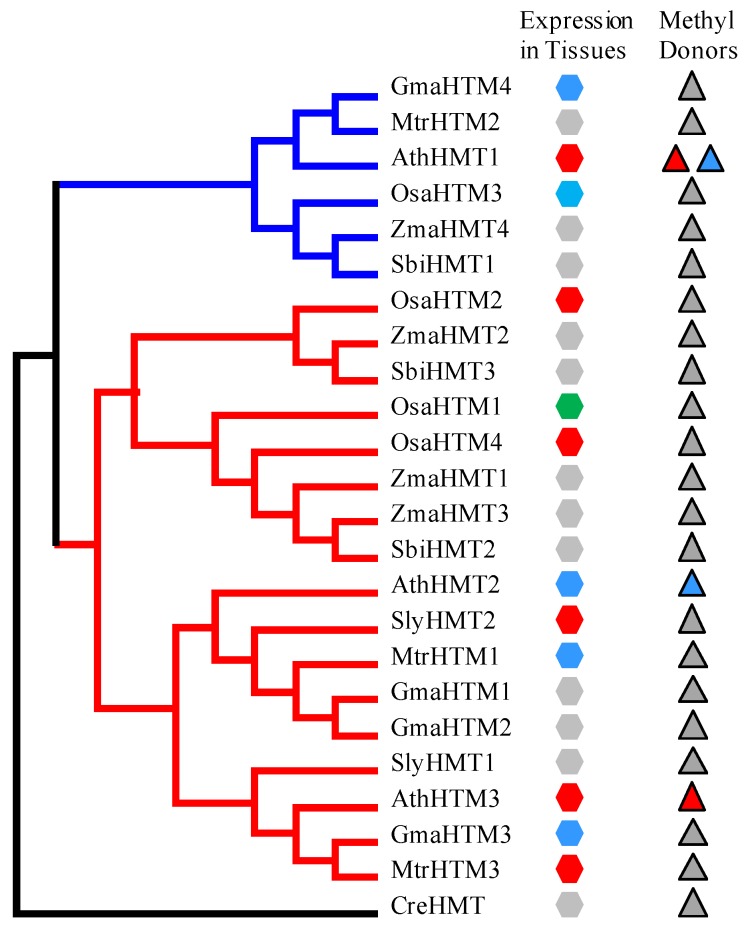
The expression patterns of *HMT* genes in represented plants. The maximum likelihood (ML) tree of *HMT*s in *Arabidopsis*, soybean, *Medicago*, tomato, rice, maize and sorghum was reconstructed with the outgroup of *CreHMT*. Class 1 and Class 2 are marked by red and blue lines, respectively. The expression patterns of tissues and methyl donors are matched to the tree. The red, green, and blue hexagons indicate that their expression were high in reproductive tissues, vegetative tissues, and fluctuant in various tissues, respectively. The gray hexagons denote their expression were unknown. The blue and red triangles indicate the methyl donors *R*,*S*-SAM and SMM, respectively. The gray triangles indicate that their methyl donors are unknown.

With the purpose of further investigating the functions of the *HMT* genes, we explored the expression of the *HMT* genes in plants. The temporal and spatial expression patterns of *AthHMT*s have been reported by Lee and Frank, in which the expression levels of *AthHMT1* and *AthHMT3* were high in reproductive organs, especially in seeds, while the expression levels of *AthHMT2* were basically similar in different tissues [7,15]. In our study, we detected the expression profiles of the *HMT* genes in different tissues in *Arabidopsis*, soybean, rice, tomato, and *Medicago* in PLEXdb (http://www.plexdb.org/index.php) [20]. The expression of *AthHMT*s was similar with previous experiments (Figure S2-1 in Additional file 3) [7,15]. The expression of *AthHMT1* and *AthHMT3* was high in seeds and flowers with the log intensity of more than 10. However, the expression of *AthHMT2* fluctuated in various tissues (Figure S2-1 in Additional file 3). In soybean, only *GmaHMT3* and *GmaHMT4* were detected in the website, and their expressions fluctuated in different tissues (Figure S2-2 in Additional file 3). As for *SlyHMT2*, the expression in fruits was higher than the vegetative organs, but in different developmental stages of fruits the expression also fluctuated (Figure S2-3 in Additional file 3). In *Medicago*, the expression of *MtrHMT1* fluctuated in different tissues, but *MtrHMT3* was up-regulated in the development of seeds (Figure S2-4 in Additional file 3). In rice, the expression of *OsaHMT1* was higher in leaves, roots, and seedlings than in the embryos and endosperms; oppositely, the expression of *OsaHMT2* and *OsaHMT4* was high in reproductive tissues and low in the vegetative tissues. As for *OsaHMT3*, the expression fluctuated in different tissues (Figure S2-5 in Additional file 3). Therefore, the data indicated that the expression of *HMT*s in tissues varied. According to their expression patterns, we divided them into different types in different tissues, namely the expression was high in vegetative tissues, high in reproductive tissues, and fluctuant in various tissues. Furthermore, we matched their tissue-specific expression patterns into their phylogenetic tree, and found that their divergence were not clade-specific (Figure 4). Nevertheless, the expression levels of *HMT*s in *Arabidopsis* and the databases have provided a hint that the expression of *HMT*s might have diverged in plants.

In order to further understand the differences of expression, we detected the promoter regions of *AthHMT*s in detail (Table S6 in Additional file 1). The detected motifs were divided into four groups according to their functions. Group 1 was involved in light response and circadian rhythm, and the numbers of involved motifs were 11 (*AthHMT1*), 13 (*AthHMT2*), and 18 (*AthHMT3*), respectively (Table S6 in Additional file 1, marked by the yellow background). The motifs in Group 2 were related to the temporal and spatial expression levels, in which the numbers of motifs in *AthHMT1* (11) were greater than those in *AthHMT2* (4) and *AthHMT3* (6), respectively. Protein interaction domains were in Group 3, mainly involving in the binding of MYB proteins. Last but not least, Group 4 was important to the response to different stresses and phytohormones, in which the kinds of motifs varied greatly among the three *AthHMT*s. The motifs of *AthHMT1* (18) and *AthHMT3* (13) numbered significantly more than those of *AthHMT2* (6), which suggested that their response degrees to different stresses might be different. Generally, the results suggested that the expression divergence of *AthHMT*s might mainly be due to their diverged promoters.

## 3. Discussion

In this study, the evolution and expression of the *HMT* genes were analyzed in plants. Similarly to *BHMT*s genes in mammals, the evolutionary pattern of the *HMT* genes also had diverged in seed plants during evolution. The diverged BHMT have different methyl donors, namely betaine or SAM [11]. In plants, the study found that the methyl donors of *AthHMT*s have also diverged [17]. AthHMT1 in Class 2 can use both *R*,*S*-AdoMet and SMM as methyl donors, while AthHMT2 and AthHMT3 in Class 1 only use *R*,*S*-AdoMet or SMM, respectively [17]. The research also proposed that the ancient function of HMTs was to repair *R*,*S*-AdoMet, and that SMM was only found in plants [17,21]. The changes of methyl donors in enzymes are likely related to their protein structures. In our study, we detected several FD I sites. Furthermore, some of these were close to the catalytic center, which might influence the catalyzing process of substrates. Therefore, the changes of methyl donors in AthHMTs might be related to these sites. Besides the methyl donors, the expression patterns of *HMT* genes in plants have diverged as well. As for the tissue-specific expression, the *AtHMT*s have diverged into two types, and are specifically highly expressed in reproductive organs (*AtHMT1* and *AtHMT3*) or are expressed in all of the organs (*AtHMT2*) [7,15]. In our study, the different tissues expressions of *HMT*s in other organisms were also investigated in PLEXdb, and the results showed that they varied in different tissues. Some *HMT*s were expressed in different tissues with similar levels or fluctuant, while some others were high expressed in seeds or leaves, and so on. However, due to the different sources of data in database, the exact expression levels and the trends of genes may need to be further explored in more organisms. Nevertheless, the expression patterns and methyl donors of *HMT* genes have diverged, and which indicated that they might have been subfunctionalized during evolution.

Previous research has confirmed that HMT is an important enzyme in the SMM cycle to supply methionine for seeds development [4,5,6,7]. Biochemical studies have also shown that the SMM cycle mainly contributes to the accumulation of Met in plant seeds [6,7,8]. In the SMM cycle, SMM is mainly synthesized in the vegetative tissues, such as leaves, and is then transported into the developing flowers and seeds through phloems, where it is reconverted back into Met by the catalysis of HMT [6,7,8]. However, how do the diverged *HMT*s function? To answer this question, we combined the expression patterns of *AthHMT*s with their methyl donors. For example, *AthHMT1* was highly expressed in seeds and flowers with the methyl donors of *R*,*S*-AdoMet and SMM. Nevertheless, the expression of *AthHMT2* was basically the same in all kinds of tissues with the methyl donors of *R*,*S*-AdoMet. As for *AthHMT3*, it was specifically highly expressed in seed organs with the methyl donor of SMM. Therefore, in view of the functional model of the SMM cycle, *AthHMT1* and *AthHMT3* might be essential to supply methionine for the development of seeds, especially *AthHMT3*, but the function of *AthHMT2* might be to repair *R*,*S*-AdoMet for the whole plant. As for *AthHMT1*, it might play two roles: supplying SMM for seeds development and repairing *R*,*S*-AdoMet. The analysis suggested that the subfunctionalization of *AtHMT*s might be related to seeds development. 

**Figure 5:**
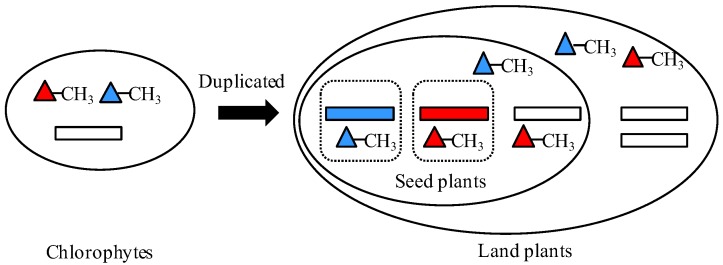
The functional model of *HMT* genes in plants. The rectangles represent *HMT* genes. The blue and red rectangles indicate the type 1 *HMT* genes in expression pattern, while the white rectangles denote other three types: type 2, type 3, and type 4. The blue and red triangles represent methyl donors *R*,*S*-SAM and SMM, respectively. The squares in dotted lines indicate different plant organs.

Generally, gene duplication supplies raw materials for the evolution and divergence of organisms. The outcomes of duplicated genes, non-functionalization, subfunctionalization, neofunctionalization, and functional redundancy largely determine the complexity and diversity of organisms’ body architecture in evolution [22,23]. Therefore, in our study the *HMT* genes contributed to the evolution of plants through subfunctionalization. According to the results, we predicted the possible evolutionary and the functional model of the *HMT* genes in plants. First, only one copy of *HMT* genes occurred in the unicellular plant algae, in which the methyl donors might be SMM and *R*,*S*-SAM. As the evolution of plants progressed, in land plants, the *HMT* genes were duplicated into two or more copies. However, these copies did not diverge until the occurrence of seed plants. In seed plants, *HMT*s diverged in their expression patterns and methyl donors, which seemed to be related to providing methionine for the development of reproductive organs, especially seeds (Figure 5). However, the real evolutionary and functional model of *HMT* genes needs to be further proved through experiments with more plants.

## 4. Materials and Methods

### 4.1. Phylogenetic Analysis of HMT Gene Family in Plants

The *HMT* genes in *A. thaliana* were used to search for *HMT* sequences in the full genomes of plants. We downloaded the *HMT* genes from Phytozome (http://www.phytozome.net/), NCBI (the National Center for Biotechnology Information, http://www.ncbi.nlm.nih.gov/), or Congenie (http://congenie.org/) with the criteria: *E*-value < 1 × 10^−5^ and an amino acid identity above 40% using the BLASTN and TBLASTN programs. In all, 75 complete *HMT* sequences were obtained in a representation of the major plant lineages (Table S1 in Additional file 1). Multiple alignments of *HMT* sequences were executed in BioEdit v 7.0.9.0 (http://www.mbio.ncsu.edu/bioedit/bioedit.html) and the Clustal X v1.81 program (http://www.clustal.org/clustal2/) with default parameters, and the manual adjustments also were used to optimize the alignments [24,25]. A ML tree was reconstructed using online PhyML (http://www.atgc-montpellier.fr/phyml/) under the JTT + G model, and Bayesian analysis (prset aamodelpr = mixed; ngen = 1,000,000) was performed with MrBayes 3.1.2 [26,27]. Resulting trees were represented using TreeView v0.4 [28].

### 4.2. Detection of Positive Selection

To estimate the selection pressures in the *HMT* gene family, the codeml program from the PAML v4.4 package (http://abacus.gene.ucl.ac.uk/software/paml.html) was performed on the basis of codon sequence alignments [19]. Branch-specific models and branch-site models were implemented [19,29]. The models tested ML estimates of ω ratios and log-likelihood (Ln L) values that were based on each examined alignment and tree topology. The assumptions were tested by the likelihood ratio test (LRT). To test for asymmetric sequence evolution, we used the two-ratio model 2 (the foreground branch is attached to a different *ω*) as compared to the branch-specific one-ratio model 0 (all of the branches are fixed to the same *ω* value). Furthermore, because selection might only occur in a few branches and sites during evolution, we used branch-site model A to detect positive selection. The *ω* value of the foreground branch was set to one for the branch-site model A as a null model. The branch-site model A was performed to estimate the selection pressure of the foreground branches and to detect the sites under positive selection with *ω* > 1 [30]. The Bayes Empirical Bayes (BEB) procedure was used to calculate the posterior probabilities when a codon was under positive selection in model A [30].

### 4.3. Divergence Analysis to Detect Sites Contributing to Functional Changes

The functional divergence of the HMT family was predicted in Diverge version 3.0 [31]. Diverge is a software system that is used to analyze the evolution and functional divergence of protein families, which performs the ML calculation of the theta (θ) type I and type II coefficients of functional divergence (FD I and FD II). The FD I and FD II are based on the variability of physiochemical properties in the protein sites, altered or radical shifts, respectively [32,33]. The sites that are under FD I mean that in one subfamily the residues are highly conserved, but are highly variable in the other, which implies that these sites have experienced altered functional constraints and different evolutionary rates [34,35]. Protein sites under FD II indicate that the amino acids in those sites are highly conserved in each subfamily, but have distinct biochemical properties, which suggest that they may be involved in functional specifications [34]. Additionally, the posterior probabilities of amino acid sites also were estimated [32,33].

### 4.4. Identification of Protein Motifs and Promoter Motifs

To detect the conserved motifs in HMT proteins, 75 complete sequences of *HMT*s were translated and uploaded to the MEME server (http://meme-suite.org/tools/meme) [36]. The server was run with the default value and choices. We conducted the search for 20 motifs arbitrarily for HMT proteins. The motifs that were retrieved by MEME are reported according to their statistical significance. The statistically significant motifs were first shown in MEME with low *E*-values. The log likelihood ratio, width, sites, and the size of the set of motifs determined their *E*-values. In our study, the significant *E*-value of the motifs identified ranges from 9.8 × 10^−1494^ (motif 1) to 4.3 × 10^−5^ (motif 16), but the remaining four motifs that were identified were not significant, and thus were not shown. The protein motifs provide important information for understanding the evolution and functions of HMT proteins.

Plant cis-acting regulatory elements were important to predict the expression of genes. To know about the gene expression of *AthHMT*s in *Arabidopsis*, we detected their regulatory elements and motifs in PlantCARE (http://bioinformatics.psb.ugent.be/webtools/plantcare/html/) [37].

### 4.5. Tertiary Structure Analysis

Three-dimensional (3D) models of AthHMT1 (AT3G25900) were generated with I-TASSER [38]. The PyMOL Molecular Graphics System was used to represent and analyze the 3D images (version 0_99rc6, 2010, https://pymol.org). We estimated the changes in stability of the point mutations in AthHMT1 at the INPS-MD (Impact of Non-synonymous mutations on Protein Stability-Multi Dimension) web server (http://inpsmd.biocomp.unibo.it/inpsSuite/default/index). The structure stability of proteins was determined by the ∆G values (ddG(change) = dG(mutant) − dG(wild-type)) [39].

## 5. Conclusions

In our study, the evolutionary history of the *HMT* gene family was studied in plants. The *HMT* genes were found have diverged into two classes. In Class 1, only the *HMT*s in seed plants were contained; in Class 2, the *HMT*s in all of the land plants were present. The exon-intron structures and protein domains showed that the *HMT*s were relatively conserved. Moreover, the selection pressure showed they were under strong purifying selection. However, the *HMT* genes had undergone type I functional divergence. The expression of *HMT* genes also showed that their expression patterns have been diverged in different developmental stages and different tissues. The results indicated that the *HMT* genes have diverged during evolution in plants, which might be related to seed development. 

## Figures and Tables

**Table 1:** Analysis of functional divergence.

FD	Clades	Coefficient θ ± SE (*p*/R)	Critical Amino Acid Sites
Type I	Class 1 vs. Class 2	0.13 ± 0.04 (*p* = 8 × 10^−4^)	44, 127*, 245*, 351*,356, 435*
	Class 1-D1 vs. Class 1-D2	0.08 ± 0.05 (*p* = 0.95)	NA
	Class 1-D vs. Class 2-D	0.20 ± 0.05 (*p* = 1.3 × 10^−5^)	75, 120*, 186*, 245, 261, 325*, 351*, 356*, 393*, 435*, 445
	Class 1-M vs. Class 2-M	0.08 ± 0.05 (*p* = 0.54)	NA
Type II	Class 1 vs. Class 2	−0.18 ± 0.23 (R = 33)	NA
	Class 1-D1 vs. Class 1-D2	0.02 ± 0.13 (R = 13)	NA
	Class 1-D vs. Class 2-D	0.05 ± 0.13 (R = 41)	NA
	Class 1-M vs. Class 2-M	−0.02 ± 0.12 (R = 11)	NA

Functional divergences (θ) for pairwise comparisons of the HMT proteins are shown as values ± standard error. Five hundred and fifty-eight sites were investigated based on posterior probability (Qk) in FD I and posterior ratio in FD II within HMT proteins. Sites with Qk > 50% are listed relative to *E. coli* HMT aa protein sequence. Sites with * meant Qk > 70%. NA meant no data was detected.

## Supplementary Materials

Supplementary materials can be found at http://www.mdpi.com/1422-0067/19/4/1248/s1.
