# Supplementary material for: Molecular Evolution and Expression Divergence of HMT Gene Family in Plants
Source: Int J Mol Sci. 2018 Apr 20;19(4):1248. doi: 10.3390/ijms19041248 (PMC5979542; doi:10.3390/ijms19041248)
Supplement: Supplementary file 1 [file ijms-19-01248-s001.zip › Additional file 2.rtf]

GmaHMT1    1 -------------------MSS-LITDLLRQTGGTAVIDGGLATELERHGADLNDP-LWSAKCLFSFP--HLIRQVHLDY
GmaHMT2    1 -------------------MSS-LITDLLRETGGSAVIDGGLATELERHGDDLNDP-LWSAKCLFSSP--HLIRQVHLDY
PvuHMT3    1 -------------------MSS-LIADLLRQSGGTAVIDGGLATELERHGADLNDP-LWSAKCLFSSP--HLIRQVHLDY
MtrHMT1    1 -------------------MSSSLISDFLHRAGGTAIIDGGLATELERHGADLNDP-LWSAKCLISIPQSHLIRQVHLDY
AbiHMT     1 -------------------MSSPLITDFLHQAGRAAVIAGGLGTELQRHGADLNDP-LWSAKCLLSCP--HLIRQVHLDY
SlyHMT2    1 ---TSDVNANS--------SSSSLVKDFLHKCGGVAVIDGGFATELERHGADLNDP-LWSAKCLLTDP--HLVRAVHLDY
VviHMT1    1 -----MGHVDL--------QPSSFISDFLLQTGGVAVIDGGLATELERHGADLNDP-LWSAKCLLSSP--HLIRTVHLDY
MdoHMT3    1 ----MASGSSA--------ETSSLMRDLLRQAGGVAVVDGGLSTELERHGADLNDP-LWSAKCLLTSP--HLIRKVHLDY
MdoHMT4    1 ----MGSSSSA--------KTPSLMRDFLRQAGGIAVVDGGLATELERHGADLNDP-LWSAKCLLTSP--HLIR------
TcaHMT2    1 ----MEALNIP--------TTPSLMRDFLRQAGGAAVIDGGLATELERHGADLNDP-LWSAKCLLTSP--HLIRSVHLDY
BraHMT4    1 ---MLTGSSKS--------EAFNSMKEFLKQTGGYAIIDGGLATEFERHGADLNDP-LWSSKCLLTSP--HIIHTVCLDY
BolHMT1    1 ---MLTRSSKS--------EAFNSMKEFLKQTGGYAIIDGGLATEFEKHGADLNDP-LWSSKCLLTSP--HIIHTVCLDY
BraHMT1    1 ---MVTGNTKA--------EAFYSMKELLKETGGYAIIDGGLATELERHGADLNDP-LWSAKCLLTSP--HLIHTVHLDY
BolHMT2    1 ---MVTGNTKA--------ETFYSMKELLKETGGYAIIDGGLATELERHGADLNDP-LWSAKCLLTSP--HLIHTVHLDY
AthHMT2    1 ----MTGNS------------FNSMKDFLKQTGGYAVIDGGLATEFERHGADLNDP-LWSAKCLVTSP--HLIHTVHLDY
AcoHMT1    1 -----MGFGIG--------NSSSAMTDFLRQSGGYAVIDGGLATELERHGADLNDP-LWSAKCLISSP--HLIKTVHLDY
GmaHMT3    1 ---MG--LEGK--------ETPSFMRDFLDKCGGCAVIDGGFATELERHGADLNDE-LWSAKCLISSP--HLVRRVHLDY
PvuHMT2    1 ---MG--LRGK--------ETPSFFRDFLDKCGGSAVIDGGFATELERLGADLNDE-LWSAKCLISSSS-HLVRRVHLDY
MtrHMT3    1 -----------------------MMKDFLNKCGGYGIIDGGFATELERHGIDLNDP-LWSAKCLFTSP--HLVRRVHLDY
MdoHMT2    1 ---------------------------MHVEACAIAYGLGGCQGELGGH------------------------LRVHLDY
TcaHMT3    1 ---MG--LEGV--------EPSVFVSDFLAKCGGYAVVDGGFATELERHGQDLNDP-LWSAKCLISSP--HLVRRVHLDY
BraHMT2    1 ---MGSLVKKG--------TSSSLMTEFLEKCGGYAVVDGGFATELERHGADIKDP-LWSAKCLITSP--HLVTKVHLDY
BolHMT4    1 ---MGSLVKEG--------TSSSSMTEFLEKCGGYAVVDGGFATELERHGADIKDP-LWSAKCLITSP--HLVTKVHLDY
AthHMT3    1 ---MGSFVKEE--------TSS-LMTDFLEKCGGYAVVDGGFATELQRHGADINDP-LWSAKCLITSP--HLVTKVHLDY
SlyHMT1    1 -------MGLK--------SESTFLGDFLRQCGGYAVIDGGLATELERHGADLNDS-LWSAKCLVSSP--HLIRRVHLDY
VviHMT3    1 ---MG--FATL--------HSPTFMADFIRQSGGYAVIDGGLATELERHGADLNDP-LWSATCLIHSP--DLIRRVHLDY
AcoHMT2    1 MGFKTVEPSSS--------TTTTLLDDFLKKSNGYAVIDGGLATELERHGANLNDP-LWSAKCLIHSP--QLITRVHLDY
ZmaHMT1    1 ---MVVTAAG---------SAEEAVRRWVDAAGGRLVLDGGLATELEANGADLNDP-LWSAKCLLSSP--HLIRKVHMDY
ZmaHMT3    1 ---MVGTAEG---------GAERAVRRWVDAAGGRLVLDGGLATELEANGADLNDP-LWSAKCLLSSP--HLIRKVHMDY
SbiHMT2    1 ---MVGTAAG---------AAEGAVRRWVDAAGGRLVLDGGLATELEANGADLNDP-LWSAKCLLSSP--HLIRKVHMDY
OsaHMT4    1 ---MVGKSG---VAEEGGAAAAAAVRRWVEAGGGRLVMDGGLATELEANGADLNDP-LWSAKCLLSSP--HLVRKVHLDY
BdiHMT2    1 ---MVVKSGGGGVGEEG--AACAAVRRWLEAGGGRLVLDGGLATELEAHGADLNDP-LWSAKCILASP--HLIRKVHLDY
OsaHMT1    1 ---MSQQGEY----------HADMMAEFLRGSGGAAVIDGGLATELEANGADLKDA-LWSARCLFTCP--DLIRKVHLDY
MacHMT4    1 ------------------------MREFLREAGGCAVIDGGLATELEAHGADLKDP-LWSANILVLCL-PMLWQSVHLDY
MacHMT2    1 ----MGFGGG---------DPEALMRDFLRELGGCAVIDGGLATELEANGADLNDP-LWSAKCLISSP--HLIRKVHLDY
MacHMT3    1 ----MGFGSG---------DPQAVMREFLHQVGGCAVIDGGLATELEANGADLNDP-LWSAKCLIGSP--HLIRKVHLDY
ZmaHMT2    1 ---MWFGGGPI--------DAAGALRGFVREAGGCAVVDGGLGTELEAHGADLHDA-LWSAKCLASAP--HLIRKVHLDY
SbiHMT3    1 -------------------MAVSYYRCFVESAK----TTRRTEITITTH-KQFCIF-LWEKK-----------AQVHLDY
BdiHMT3    1 ---MGRGDGYD--------DAAGALRGLVREAGECLVLDGALGTELEAHGADLQDE-LWSASCLVSAP--HIIRKVHLDY
OsaHMT2    1 ---MGRGGDVD--------GAAGALRRFVREAGGCAVVDGGLATELEAHGADLHDE-LWSASCLVSAP--HLIRKVHLDY
BraHMT3    1 -----------MGLEKK----SALLEDLIEKCGGCAVVDGGFATQLEIHGAAINDP-LWSAVSLIKDP--ELIKRVHMEY
BolHMT3    1 -----------MGLEKK----SALLEDLIEKCGGCAVVDGGFATQLEIHGAAINDP-LWSAVSLIKDP--ELIKRVHMEY
AthHMT1    1 -----------MVLEKK----SALLEDLIKKCGGCAVVDGGFATQLEIHGAAINDP-LWSAVSLIKNP--ELIKRVHMEY
VviHMT2    1 -------------MGKT----SSLLEDLIEKAGGCAVVDGGFATQLEIHGATINDP-LWSALCLIKDP--DLIKRVHLEY
TcaHMT1    1 -----------MGFGKG----TSLLEDLIDKAGGCAVIDGGFATQLETHGASINDP-LWSALCLIKDP--DLIKQVHLEY
GmaHMT4    1 ----------------MK---RQMLHDLIENAGGCAVTDGGFATQLEKHGASINDP-LWSAIYLIKDP--HLIKQVHLEY
PvuHMT1    1 -------------MKSEK---RQMLHHLIENAGGCAITDGGFATQLEKHGASINDS-LWSAIYLIKDP--NLIKQVHLEY
MtrHMT2    1 -------------MKGEK---KSLLQDLIENSGGCVVTDGGFATQLEKHGAFINDP-LWSAICLIKQP--HLIKKVHMEY
MdoHMT1    1 -----------MGLKKAF---TSSLEDVIEKAGGCAVVDGGFATQLERHGAAINDP-LWSAVCLIKQP--DLIKRVHLDY
AcoHMT3    1 -----------MGFEKKTQKAMSLLEEVIEKAGGCAVIDGGFATQLEKHGASINDP-LWSALCLIKDP--QLIKKVHLEY
ZmaHMT4    1 ---------------------MGVLEDLVARAGGCAVIDGGFATQLEALGADINDP-LWSAACLITRP--HLVKEVHMQY
SbiHMT1    1 ---------------------MGALEELVAKAGGCAVIDGGFATQLEALGADINDP-LWSAACLITRP--HLVKEVHMQY
OsaHMT3    1 --------------------MAVAVEEIVRRAGGCAVIDGGFATQLEALGADINDP-LWSAACLITKP--HLIKEVHMQY
BdiHMT1    1 --------------------MAGVVEELVKKAGGCAVIDGGFATQLEALGADINDS-LWSAACLITKP--HLIKEVHMQY
MacHMT1    1 -----------------MGSRTGLVEELIERNCGVAVIDGGLATQLEALGADINGP-LWSARCLISDP--HLIKQVHLQY
MpoHMT     1 -------MGCVDKVLALGAHPFNRVDLLLRNSGGSAVIDGGFAVQLEHHGAEVKNP-LWTALCLITSP--DLVSRVHWDY
SmoHMT2    1 ------------MGSDARPGGFDCLEELVRHKG-CVVKDGGFATQLEKHGALLNDP-LWSALCLITNP--GLIAKVHWEY
PpaHMT1    1 ----MGRAVDIPEDKLN------ALSELLKTAGGCVTTDGGFATQLERHGADINDP-LWSASCLITIP--ELVRKVHREY
PpaHMT3    1 ----MGHTLNLHHDTFQEPERSETITELLKQAGGCVVTDGGFATQLERHGADINDP-LWSALCLITMP--HLIRTVHKEY
PpaHMT2    1 ----MGRTIDVPEDTLQESETSNVVLELLKQAGGCVVTDGGFATQLERHGANINDP-LWSAVCLITMP--DLIRKVHREY
SfaHMT1    1 ----MGLAVHKNSEPVIASEAFGRLNELVKQAGGCVAIDGGFATQLERHGADINDS-LWSALCLITMP--ELIRKVHREY
SfaHMT2    1 -------MGRALHLLSEEPESFKAFADLLKRAGGCVAIDGGLATQLEQHGADIKDP-LWSALCLITSP--DLIRKVHKEY
SmoHMT1    1 -----------------MGAGKNKLEELLESSGGCAVLDGGLATQLEHCGADLNDP-LWSALCLITRP--QLIQKVHWDY
VcaHMT     1 --------------MGPANNQCSMLSSLLTNTGGVLILDGAQGTELERRGVHLGGSKLWSAQLLIDDP--DLIRTIHLDY
CreHMT     1 --------------------MHNPLEPLLAEQG-VVILDGAQGTELERRGIDIGGSKLWSAQLLIDDP--DTVQAIHLDY
EcoHMT     1 ------------------MSQNNPLRALLDKQD-ILLLDGAMATELEARGCNLADS-LWSAKVLVENP--ELIREVHLDY
PabHMT1    1 -------------------------------------------------------------------------MQVHLEY
PabHMT2    1 ----MSSAP----------KSVKVIES-----------------------GKTND------------------HKVHQEY
PtaHMT1    1 ---MKFGSG----------ERMKVLEEFLQQVGGFGVIDGGLATQLESHGADLNDP-LWSGRCLIESP--HLIQEVHQEY
PtaHMT2    1 -----------MGERIVGLGRWGALEGLIKRAGGCAVVDGGLATQLENHGANINDP-LWSALCLITNP--NLIKQVHLEY
AtrHMT1    1 ---MG--LEEK-------------MEEFLQKVGGSAVIDGGFATELERHGADLNDP-LWSAKCLITSP--HLIRQVHLDY
AtrHMT2    1 --------------------MAEVVEELIERAGGCAVIDGGLATRLEERGADINHP-LWSALCLINDP--HLIKQVHLEY
SpoHMT1    1 -----------------MGMGKGPVEDLIERAGGCAVIDGGFATQLELEGADISDP-LWSAVCLISNP--NLITRVHLQY
SpoHMT2    1 ---MAAMGVDP----------SAVLRDFLRESGGCAIIDGGLATELEANGADLNDP-LWSAKCLIASP--ELIRKVHLDY
ZomHMT     1 ---MEIQGCRED-------RTSSLMVDFVKDCGGIAIIDGGLATELEANGADLNDP-LWSAKCLFNSP--HLIQKVHLDY
                                                

             
GmaHMT1   58 LENGADIIITASYQATIQGFKAKGYSDEESEALLRSSVEIAREAREVYYKNCAGCRSGD---------------------
GmaHMT2   58 LENGADIIITASYQATIQGFKAKGYSDEESEALLKRSAEIAFEAREVYDKNCAGCCSGD---------------------
PvuHMT3   58 LENGADIIITASYQATIQGFKAKGYTEEESEAMLRRSVEIAREARELYYKECS---SGD---------------------
MtrHMT1   61 LENGADIITTASYQATIQGFKEKGFSNEESENMLRRSVEIACEARDLYYERCAACSSGK---------------------
AbiHMT    59 LENGADIIITASYQATIQGFKAKGFSDEEGEALLRRSVEIAREARDLYYQRCAESSSDN---------------------
SlyHMT2   67 LEAGADIILSSSYQATIQGFKAKGYSIEESESLLKRSVEIACEARDVYYKRCRESSSDQ---------------------
VviHMT1   65 LEAGADIIITASYQATIQGFEARGFSRGESEALLRKSVEIACEARKM---------------------------------
MdoHMT3   66 LEAGADIIITASYQATIQGFKAKGFSTEESESLLRKSVEIAREARDIYYDRCTQCSSAD---------------------
MdoHMT4   60 -----TLKVLVAMQATIQGFEAKGYSTEESEALLRKSVEIAREARDVYYDRCTQCSSAD---------------------
TcaHMT2   66 LEAGADIIITASYQATIQGFEAKGFSREESETLLKKSVEIALEARDIYYERCNKNSCDG---------------------
BraHMT4   67 LEAGADIIASASYQATIQGFETKGFSIEKSEYLLRKSVEIACEARSTYYDKCKATSSSS---------------------
BolHMT1   67 LEAGADIIASASYQATIQGFETKGFSREKSEYLLRKSVEIACEARSTYYDKCKATSSSS---------------------
BraHMT1   67 LEAGADIISSASYQATIQGFEAKGYSIEKSESLLRKSVEIACEARNTYYDKCKDD-------------------------
BolHMT2   67 LEAGADIISSASYQATIQGFEAKGYPIEKSESLLRKSVEIACEARSTYYDKCKDD-------------------------
AthHMT2   62 LEAGADIISSASYQATIQGFEAKGFSREESESLLKKSVEIATEARNSYYDKCGTSSS-----------------------
AcoHMT1   65 LEAGADIIITASYQATIQGFESKGMSRDEGEALLKRSVEIACEARDTYYERCAEHHLSE---------------------
GmaHMT3   65 LDAGANIILTASYQATIQGFEAKGFSREEGETMLRRSVEIAREAREIYYDRCTKDSSD----------------------
PvuHMT2   66 LDAGANIILTASYQATVQGFEAKGFSREEGVTMLKRSVEIAREAREIYHDRCTKDSSD----------------------
MtrHMT3   55 LDSGANIILTSSYQATIQGFEAKGFSKEEGQALLRRSVELAREARDIYYDRCTKDSFD----------------------
MdoHMT2   30 LDAGANIILTASYQATIQGFEAKGFSKEEAKDLLRKSVEIAIEAREIYYDKFQ---------------------------
TcaHMT3   65 LDAGANVIITASYQATIQGFEAKGLSTEEAETLISRSVEIACEARQIYHDKCTKDSWD----------------------
BraHMT2   67 LESGANIIITASYQATIQGFVAKGLSVGEAESLLRRSVELSCEAREIFYNRCNKGSWD----------------------
BolHMT4   67 LESGANIIITASYQATIQGFVAKGLSVGEAESLLRRSGELSCEAREIFYNRCNKGSWD----------------------
AthHMT3   66 LESGANIIITASYQATIQGFVAKGLSVGEAENLLRRSVEITYEAREIFYNRCTKGSWD----------------------
SlyHMT1   63 LEAGANIIISSSYQATLQGFEAKGISREEGEALLKRSVEIACEARNIYNDRASKGSWDD---------------------
VviHMT3   65 LEAGASIIITASYQATIQGFEAKGLSREEAEVLLRRSVEIACEARDIYHERCAKGTC-----------------------
AcoHMT2   70 LEAGASIIITASYQATIQGFEAKGLSRQESEALLKKSVEIALEARDIFYGSHTERSSDD---------------------
ZmaHMT1   66 LEAGANIIITASYQATIQGFESKGFSKEQSENLLTKSVEIALEAREMFLK------------------------------
ZmaHMT3   66 LEAGANIIITASYQATIQGFESKGFSKEQSENLLTKSVQIALEAREMFLK------------------------------
SbiHMT2   66 LEAGANILITASYQATIQGFESKGFSKEQSENLLTKSVEIALEAREMFLK------------------------------
OsaHMT4   72 LEAGANIIITASYQATIQGFESKGFSKEQSEDLLAKSVEIAREARDMFLK------------------------------
BdiHMT2   73 IEAGANIIITASYQATIQGFESKGFSKQQGEDLLTKSVKVAQEAREMFLK------------------------------
OsaHMT1   65 LEAGASVLITGSYQATIQGFLSKGFSQEESESFLRRSVELACEARAIYLEKCSNGSDE----------------------
MacHMT4   55 LEAGANILITASYQATIQGFESRGFSTEESEALLRRSVELACEAREIFQGRRLRASDR----------------------
MacHMT2   65 LEAGANIIITSSYQATIQGFESRGFSAEESEALLQKSAEIACEARDIFYKGCS----K----------------------
MacHMT3   65 LEAGAHVIITSSYQATIQGFKSRGFSQEESEDLLRRSVQIAREARDIFYNDCL----R----------------------
ZmaHMT2   67 LEAGADVIISASYQATIEGFQSRGFSRDESEELLRRSVHVAQEARRVFAAEGDR-SSR----------------------
SbiHMT3   45 LEAGADVIISASYQATIEGFQSRGFSRDESEELLRRSVHVAQEARRVFVAEGDVDSSRS---------------------
BdiHMT3   67 LEAGANIITTASYQATLQGFQSRGLSSEQSETLLRRSVEIAQEARAIFVEGRSKGPYAG---------------------
OsaHMT2   67 LDAGANIITSASYQATIQGFQARGLSRERSEALLRRSVHIAQEARAIFAEGWSKGPYA----------------------
BraHMT3   63 LEAGADIVVTSSYQATIPGFLSRGLSMEESESLLQKSVKLAVEARDRFWD------------------------------
BolHMT3   63 LEAGADVVVTSSYQATIPGFLSRGLSMEESESLLQKSVKLAVEARDRFWD------------------------------
AthHMT1   63 LEAGADIVVTSSYQATIPGFLSRGLSIEESESLLQKSVELAVEARDRFWE------------------------------
VviHMT2   61 LEAGADILVTSSYQATIPGFLSKGLSIEEGELLLERSVRLAVEARDKFWD------------------------------
TcaHMT1   63 LEAGADILVTSSYQATIPGFLSRGLSLEEAESLLEKSVQLAVEARDKFWD------------------------------
GmaHMT4   59 LEAGADILVTSSYQATLPGFSSKGLSIEEGESLLEKSVKLAVEARDGFWN------------------------------
PvuHMT1   62 LEAGADILVTSSYQATLPGFASKGLSIEEGESLLERSVKLAVEARDTFWS------------------------------
MtrHMT2   62 LEAGADILVTSSYQATIPGFLSKGLSIEEGESLLQRSVKLAVEARDSFWS------------------------------
MdoHMT1   64 LEAGADILITSSYQATIPGFLSRGLSIEQGELLLKKSVKLAVEARNSFWD------------------------------
AcoHMT3   67 LEAGADILVTSSYQATIPGFLSRGLTMEEGESLLRKSVKLATEARDKFWE------------------------------
ZmaHMT4   57 LEAGADVIISSSYQATIPGFIARGMSVAEAEDLLRTSVKLANEARDEFWK------------------------------
SbiHMT1   57 LEAGADIIISSSYQATIPGFLARGMSVDEAEDLLRTSVKLAVEARDEFWK------------------------------
OsaHMT3   58 LEAGADVIISSSYQATIPGFLARGMLLEEAEGLLRRSIELALEARDEFWK------------------------------
BdiHMT1   58 LEAGADVIISSSYQATIPGFLARGLRQEEAEGLLRTSVHLALEARDEFWK------------------------------
MacHMT1   61 LEAGADILVTSSYQATIPGFLSKGFSIEEGELLLQQSVKLALEARDNFWK------------------------------
MpoHMT    71 LEAGAQIVLTSSYQATLLGFESRGYTREQGKEFLRRSVTLACEARDKFWNEYQQR---V---------------------
SmoHMT2   65 LESGAEVLVTSSYQATLQGFQSRGISLEESEALLRKSVTLACEARDRFWR------------------------------
PpaHMT1   68 LEAGAGVISTASYQATIQGFQSRGLSTNEAEDLLQRSVRIAQEERDRVWKESQNR---E---------------------
PpaHMT3   74 LQAGASVISTASYQATIQGFQSRGLSTKEAEDLLQTSVRIAQEERDSFWKEYQNK---V---------------------
PpaHMT2   74 LEAGAAVISTASYQATIQGFEMRGLSTKDSEDLLQLSVRIAREERDRFWKEYQNK---V---------------------
SfaHMT1   74 LEAGARIISTASYQATIQGFQSKGFSKEEGETFLRRSVEIACQERDLFWEEHQQQ---V---------------------
SfaHMT2   71 LEAGAEVLVTASYQATLQGFEARGLTQAEGEALLQKSVTIACEERDRFWDQYIDR---L---------------------
SmoHMT1   61 LEAGADILVSSSYQATVQGFVSKGLSEKEGEEMLKKSVAIACQVRDKFWDKVK---------------------------
VcaHMT    65 LRAGSDVITTFTYQASIQGFADAGMDARMGATLLNRAVDLAESARTAFLDEQRQ--------------------------
CreHMT    58 LRSGSDVITTFTYQASLPGFAEAGVDAAHAGRLLNLAVDLAEAARAHFMREQEQERGQEQEQGKEQDQGKEPGHQGQGQE
EcoHMT    59 YRAGAQCAITASYQATPAGFAARGLDEAQSKALIGKSVELARKAREAYLAENPQ--------------------------
PabHMT1    8 LGAGAEVLVTSSYQATLPGFTSRGLSIEQGESLLQKSVKLACEARDSFWEN-------V---------------------
PabHMT2   26 LEAGAEIIITASYQATIQGFESRGLSITEGEALLRRSVEIACEARDQFWKKCAESLNG----------------------
PtaHMT1   65 LEAGAEIIITASYQATIQGFESKGFSVTEGEALLRKSVEIACEAREQFWKKCAEILNG----------------------
PtaHMT2   67 LGAGAEVLGTSSYQATLPGFTSRGFSIEQGESLLQKSVKLACEARDSFWEN-------V---------------------
AtrHMT1   60 LEAGAEIIITSSYQATIQGFKSKGFSEKEAEELLVKSVHIACEARDIYWERHSKSSCNK---------------------
AtrHMT2   58 LEAGADIIVTSSYQATLPGFLSNGISKEEGELLLQRSVKLACEARDTFWE------------------------------
SpoHMT1   61 LEAGADIIVTSSYQATIPGFLARGLSIEGGERLLQKSVQLALDARDSFWK------------------------------
SpoHMT2   65 LEAGADILITASYQATIQGFQSKGFSVEQSEALLRKSVTVACEAREIFYKKHTSYLCN----------------------
ZomHMT    68 LEAGANILITSSYQATIQGFESRGFSKDQSEGLLRKSVEVACEARRIFQEKHVKASKT----------------------
                 

        
GmaHMT1  117 ------------------GDDDGRILKQ-------------------------RPILVAASVGSYGAYLADGSEYSGDYG
GmaHMT2  117 ------------------GEDDGRILKQ-------------------------RPILVAALVGSYGAYLADGSGYSGDYG
PvuHMT3  114 ------------------G-ADGKILKQ-------------------------RPILIAASVGSYGAYLADGSEYSGDYG
MtrHMT1  120 ------------------N-ADDRILKQ-------------------------RPILIAASVGSYGAYLADGSEYSGNYG
AbiHMT   118 ------------------G-DDSRILKQ-------------------------RPILIAGSVGSYGAYLADGSEFSGNYG
SlyHMT2  126 ------------------S-TDGKVLKQ-------------------------RPILVAASVGSYGAYLADGSEYSGEYG
VviHMT1  112 ------------------------ILKH-------------------------RPILVAASVGSYGAYLADGSEYSGIYG
MdoHMT3  125 ------------------S-GNGRILKR-------------------------RPILVAASVGSYGAYLADGSEYSGNYG
MdoHMT4  114 ------------------S-ANGRILKR-------------------------RPILVAASVGSYGAYLADGSEYSGDYG
TcaHMT2  125 ------------------L-GDGRILKI-------------------------RPILVAASVGSYGAYLADGSEYSGDYG
BraHMT4  126 ------------------DIVDDKILKT-------------------------RPILVAASVGSYGAYLADGSEYSGIYG
BolHMT1  126 ------------------DIVDDKILKT-------------------------RPILVAASVGSYGAYLADGSEYSGIYG
BraHMT1  122 --------------------NNK-FLKK-------------------------RPILVAASVGSYGAFLADGSEYSGIYG
BolHMT2  122 --------------------DDKKILKK-------------------------RPILVAASVGSYGAFLADGSEYSGIYG
AthHMT2  119 --------------------MDDKILKK-------------------------RPILVAASVGSYGAYLADGSEYSGIYG
AcoHMT1  124 ------------------GIKNDKILKK-------------------------RPILVAASVGSYGAYLADGSEYDGNYG
GmaHMT3  123 -------------------FMRDERYRK-------------------------RPILIAASVGSYGAYLADGSEYVGDYG
PvuHMT2  124 -------------------FVRNERFRK-------------------------RPILIAASVGSYGAYLADGSEYVGDYG
MtrHMT3  113 -------------------FIRDERYRS-------------------------RPILIAASVGSYGAYLADGSEYTGDYG
MdoHMT2   83 --------------------------SR-------------------------RPVLVAASIGSYGAYLADGSEYSGNYG
TcaHMT3  123 -------------------FLVDGNITR-------------------------RPVLVAASVGSYGAYLADGSEYTGNYG
BraHMT2  125 -------------------FDHAGKASR-------------------------RPVLVAASVGSYGAYLADGSEYSGVYG
BolHMT4  125 -------------------FDHAGKASR-------------------------RPVLVAASVGSYGAYLADGSEYSGVYG
AthHMT3  124 -------------------FAYAGKASR-------------------------RPILVAASVGSYGAYLADGSEYSGIYG
SlyHMT1  122 -------------------FIDGAGLKR-------------------------NPVLVAASVGSYGAYLADGSEYSGIYG
VviHMT3  122 -------------------------LEQ-------------------------RPILVAASVGSYGAYLADGSEYSGHYG
AcoHMT2  129 -------------------FGNERTHQR-------------------------RPVLVAASIGSYGAYLADGSEYSGDYG
ZmaHMT1  116 ------------------EHLEKSTPIQ-------------------------HPVLVAASLGSYGAYLADGSEYSGDYG
ZmaHMT3  116 ------------------EHLEKSTPIQ-------------------------HPILVAAALGSYGAYLADGSEYSGDYG
SbiHMT2  116 ------------------EHLEKSIPIQ-------------------------HPILVAASIGSYGAYLADGSEYSGDYG
OsaHMT4  122 ------------------EHSDR--PIQ-------------------------HPILVAASIGSYGAYLADGSEYSGDYG
BdiHMT2  123 ------------------EHPDQSTPMQ-------------------------HPILVAASIGSYGAYLADGSEYSGDYG
OsaHMT1  123 -------------------AKDVTKYRK-------------------------RPILIAASVGSYGAYLADGSEYSGDYG
MacHMT4  113 ------------------CSKDGISSKQ-------------------------HPVLIAASIGSYGAYLADGSEYSGNYG
MacHMT2  119 ------------------GFENHNETKK-------------------------HTVLVAASIGSYGAYLADGSEYSGEYS
MacHMT3  119 ------------------GYGGQSDIKK-------------------------HTVLVAASIGSYGAYLADGSEYSGHYG
ZmaHMT2  124 -------------------------RGR-------------------------PPALVAASVGSYGAYRADGSEYSGDYG
SbiHMT3  104 -------------------R-RERERER-------------------------PPVLVAASIGSYGAYRADGSEYSGDYG
BdiHMT3  126 -------------------RENDGSRER-------------------------RPVLVAASVGSYGAYLADGSEYTGDYG
OsaHMT2  125 ---------------------NHRSSPR-------------------------RPVLVAASIGSYGAYLADGSEYTGDYG
BraHMT3  113 ------------------KVSKTSGHSY-------------------------NRALVAASIGSYGAYLADGSEYSGSYG
BolHMT3  113 ------------------KVSKTSGHSY-------------------------NRALVAASIGSYGAYLADGSEYSGSYG
AthHMT1  113 ------------------KVSKVSGHSY-------------------------NRALVAASIGSYGAYLADGSEYSGHYG
VviHMT2  111 ------------------VTKRVPGHGY-------------------------NRALVAASIGSYGAYLADGSEYSGCYG
TcaHMT1  113 ------------------AVGSVPGNSY-------------------------NRALVAASIGSYGAYLADGSEYSGCYG
GmaHMT4  109 ------------------SAIINPGNKY-------------------------RRALVAASIGSYGSYLADGSEYSGCYG
PvuHMT1  112 ------------------SAKRNPGNKY-------------------------RRALVAASIGSYGAYLANGSEYSGCYG
MtrHMT2  112 ------------------SAKRNPGNKY-------------------------RRALVAASIGSYGAYLADGSEYRGLYG
MdoHMT1  114 ------------------ALKVTPDHRY-------------------------NRALVAASIGSYGAYLADGSEYSGCYG
AcoHMT3  117 ------------------ANKKIPENRY-------------------------NRALVAASIGSYGAYLADGSEYSGCYG
ZmaHMT4  107 ------------------STLRKSKPIY-------------------------NRALVAASIGSYGAYLADGSEYSGSYG
SbiHMT1  107 ------------------SALRKAKPIY-------------------------NRALVAASVGSYGAYLADGSEYSGSYG
OsaHMT3  108 ------------------STLRKSKPVY-------------------------NRALVAASIGSYGAYLADGSEYSGSYG
BdiHMT1  108 ------------------STLTKPKPIY-------------------------NRALVAASIGSYGAFLADGSEYSGSYG
MacHMT1  111 ------------------LNADN---YY-------------------------IRALVAASIGSYGAYLADGSEYSGHYG
MpoHMT   127 ------------------QKHEAAPGQY-------------------------CRALVGASIGSYGAYLADGSEYSGDYG
SmoHMT2  115 ------------------TKRAQGAERF-------------------------NRPLVAASIGSYGAFLADGSEYSGDYG
PpaHMT1  124 ------------------HARTARAGSN-------------------------LRALVAASIGSYGAYLADGSEYSGDYG
PpaHMT3  130 ------------------RAGTAHAGLY-------------------------QRALAAASVGSYGAYLADGSEYSGDYG
PpaHMT2  130 ------------------HTGPGQAGSY-------------------------HHALVAASIGSYGAYLADGSEYSGDYG
SfaHMT1  130 ------------------QEQNVGLGKV-------------------------QRALVAASIGSYGAYLADGSEYSGDYG
SfaHMT2  127 ------------------RLGLAEPGMY-------------------------RRALIAASIGSYGAFLADGSEYSGDYG
SmoHMT1  114 ------------------QNNSSGEIRY-------------------------NRALVAASIGSYGAYLADGSEYSGQYG
VcaHMT   119 --------------QHEQPPPHHQQRVR---------------------------PLIAFSSGSYGAYLADGSEFRGDYA
CreHMT   138 QGKEREQGKEQGEEQEQQESGQHGQRLRGAGSSAAGGGSGSGDSRRHAAAGRRRRPLIAYSCGSYGAYLADGSEFQGDYA
EcoHMT   113 -------------------AG---------------------------------TLLVAGSVGPYGAYLADGSEYRGDYH
PabHMT1   60 ------------------QNCESLDKKC-------------------------NRALVAASIGSYGAYLADGSEYSGDYG
PabHMT2   84 ------------------SVDDAQIPKV-------------------------RPILVAASVGSYGAYLADGSEYSGDYG
PtaHMT1  123 ------------------SVDDAQIPKV-------------------------RPILVAASVGSYGAYLADGSEYSGDYG
PtaHMT2  119 ------------------QNCETLDKKY-------------------------NRALVAACIGSYGAYLADGSEYSGDYG
AtrHMT1  119 -------------------FGNGNVLER-------------------------DPILVAASIGSYGAYLADGSEYSGDYG
AtrHMT2  108 ------------------FLQRMPGHRH-------------------------SCALVAASVGSYGAYLADGSEYSGNYG
SpoHMT1  111 ------------------AHLRAPRRGY-------------------------HRALVAASVGSYGAYLADGSEYSGRYG
SpoHMT2  123 ------------------SSSGGMPFKE-------------------------RSILVAASIGSYGAYLADGSEYSGHYG
ZomHMT   126 ------------------P----------------------------------HQVLIAASIGSYGAYLADGSEYSGDYG
                                                                                  

     
GmaHMT1  154 DAIT-VETLKDFHRRRVQILAD-SGADLLAFETVPNKLEAEAY----------AQLLEEEDIKIPAWFSFNSKDGVNVVS
GmaHMT2  154 DAIT-VE-----IRRRVQILAD-SSADLLAFETVSNKLEAEH--------------------------GFLLTHGVNVVS
PvuHMT3  150 DAIT-KTTLKDFHRRRVQILAD-SGADLLAFETVPNKLEAEAY----------AQLLEEENIQIPAWFAFNSKDGVNVVS
MtrHMT1  156 DAIT-LKTLKDFHRRRVQVLAD-ASADLLAFETIPNKIEAQAF----------AELLEEENIKVPAWFCFNSKDGINVVS
AbiHMT   154 DAIK-SETLKDFHRRKVQILAD-SGVDLLAFEAVPNKLEAQAY----------ADLLEEENIITPAWFAFTSKDGNNVVS
SlyHMT2  162 DAVD-LKFLKDFHRRRVHLLAN-SGADLIAFETVPNKLEAQAF----------VELLKEEDIKTPAWLSFNSKDGVNVVS
VviHMT1  143 DEIT-VETLKDFHRRRVQILAD-AGADLIAFETVPNKLEAQAY----------AELLEEENIKIPAWFSFNSKDGVHVVS
MdoHMT3  161 EAMT-LGRLKDFHRRRVQILAE-SGPDLLAFETVPNKLEAQAY----------AELLEEENMQLPAWFSFNSKDGINVVS
MdoHMT4  150 EAMT-LGRLKDFHRRRVQVLAA-SGPDLLAFETVPNKLEAQAY----------AELLEEENMQLPAWFSFNSKDGINVVS
TcaHMT2  161 DAMT-IEALKEFHGRRVQVLAE-AGPDLIAFETVPNKIEAQAY----------VELLQEEDIKIPAWFSFNSKDGINVVS
BraHMT4  163 DLIT-LETLKDFHRRKLQVLGE-SGADIIAFETIPSKLEAQAF----------AELLDEGDVKISGWFSFNSNDGVNVVS
BolHMT1  163 DLIT-LETLKDFHRRKLQVLGE-SGADIIAFETIPSKLEAQAF----------AELLDEGDVKISGWFSFNSNDGVNVVS
BraHMT1  156 DLIT-LETLKDFHRRRVQVLAE-SGADIIAFETIPNKLEAQAF----------AELLDEGVVKIPGWFSFNSKDGVNVVS
BolHMT2  157 DLIT-LETLKDFHRRRVQVLAE-SGADIIAFETIPNKLEAQAF----------AELLDEGVAKIPGWFSFNSKDGVNVVS
AthHMT2  154 DSIT-LEKLKDFHRRRLQVLAE-SGADLIAFETIPNKIEAQAF----------ADLLEEGDVKIPGWFSFNSKDGVNVVS
AcoHMT1  161 DAVT-LSTLKEFHRRRVQVLAE-AGPDLIAFETIPNKLEAQAF----------AELLEEDGINIPAWFAFNSKDGVNVVS
GmaHMT3  159 DAVT-VQTLKDFHRERVKILVE-AGADLIAFETIPNKLEAQAY----------AELLEEEGIETPAWFSFSCKDESNVVS
PvuHMT2  160 DAVT-VQTLKDFHRERVKILVD-AGADLIAFETIPNKLEAQAY----------AELLDEEGIETPAWFSFSCKDECNVVS
MtrHMT3  149 DAIT-VHTLKDFHRERVKILVD-AGADLIAFETIPNKLDAQAY----------AELLEEEGIEIPAWFSFSCKDENNVAS
MdoHMT2  112 DAIT-VETLKDFHRERVQILAN-SGADLIAFETIPNKIEAKAY----------AELLDEEGIEIPAWFSFSSKDGINVVS
TcaHMT3  159 DSVT-LESLKDFHRRRLQILAT-SGADLIAFETIPNKLEAQAY----------AELLEEEGIDIPSWFSFTSKDGINVVS
BraHMT2  161 DSVS-KESLKDFHRRRVQILAK-SGADLIAFETIPNKLEAEAY----------VDLLEEDGIDIPAWFAFTSKDGVTVPS
BolHMT4  161 DSVS-KESLKDFHRRRVQILAK-SGADLIAFETIPNKLEAEAY----------VDLLEEEGIDIPAWFAFTSKDGVTVPS
AthHMT3  160 DSVS-KETLKDFHRRRVQILAK-SGADLIAFETIPNKLEAEAY----------ADLLEEEDIDIPAWFSFTSKDGVSVPR
SlyHMT1  158 DAIT-VKALKDFHRRRVQVLAD-SGADLIAFETTPNKIEAQAY----------AEILEEEAINVPVWFSFSSKDGINVAS
VviHMT3  152 AAVT-LETLKDFHRRRVQVLAE-SGADLIAFETIPNKLEAKAY----------AELLDEENIKIPAWFSFTSLDGINVVS
AcoHMT2  165 DAVT-LSTLKDFHRRRVQVLAE-SGADLIAFETIPNKLEAQAY----------SEILEEENINVPAWYSFNSKDGVNVVN
ZmaHMT1  153 EAGT-KEFLKDFHRRRLQVLAE-AGPDLIAFETIPNKLEAEAY----------VELLEECNINIPAWFSFNSKDGVHIVS
ZmaHMT3  153 EAGT-KEFLKDFHRRRLQVLAE-AGPDLIAFETIPNKLEAQAY----------VELLEECNINIPSWLSFNSKDGVHVVS
SbiHMT2  153 EAGT-KEFLKDFHRRRLQVLAE-AGPDLIAFETIPNKLEAQAY----------VELLEECNINIPAWLSFNSKDGVHIVS
OsaHMT4  157 EAGT-LEFLKDFHKRRLEVLAE-AGPDLIAFETIPNKLEAQAY----------VELLDECNISIPAWFSFNSKDGVHIVS
BdiHMT2  160 EAGT-LEFLKDFHRRRLQVLAE-AGPDLIAFETIPNKLEAQAY----------VELLDECNISIPSWFSFNSKDGVNVVS
OsaHMT1  159 NEGT-LEFLKNFHLRRLQVLAE-AGPDVIVFETIPNKIETQAY----------VELLEECKLRIPAWFGFTSKDGVNVVS
MacHMT4  150 LEVT-LETLKDFHRRRLQVLSE-AGADIIAFETIPCKIEAQAY----------IELLQECNIKIPVWFSFTSNDGINIVS
MacHMT2  156 KEMT-LEKLKNFHRRRVEVLAE-SGADLIAFETIPNKLEAQAY----------AELLEENNTRTPAWFSFNSKDGVNVVS
MacHMT3  156 KDMT-IENLKDFHRRRLEVLAE-GGADLIVFETIPNKLEAQAY----------AELLEEDNISIPAWFSFNSKDGVNVVS
ZmaHMT2  154 KSMT-KEDLKNFHRRRLQVLAG-AGPDLIAFETIPNKLEAQVY----------AELLEENGIRIPAWFSFTSKDGVNAAS
SbiHMT3  139 KSVT-KEALKDFHRRRLQVLAG-AGPDLIAFETIPNKLEAQAY----------AELLEENGIRIPAWFSFTSKDGVHAAS
BdiHMT3  162 RSVT-KEALKNFHRRRLQVLAD-AGPDLIAFETIPNKLEAQAY----------SELLEENDIRIPAWFSFTSKDGANAAS
OsaHMT2  159 ISVT-KETLKSFHRRRLQVLAD-AGPDLIAFETIPNKLEAQA-------------------------------------S
BraHMT3  150 EDVT-LDKLKDFHRRRIQVLVE-ASPDLLAFETIPNKLEAQAC----------VELLEEENVQIPAWICFTSVDGENAPS
BolHMT3  150 EDVS-LDKLKDFHRRRIQVLVE-ASPDLLAFETIPNKLEAQAC----------VELLEEENVQIPAWICFTSVDGENAPS
AthHMT1  150 ENVS-LDKLKDFHRRRLQVLVE-AGPDLLAFETIPNKLEAQAC----------VELLEEEKVQIPAWICFTSVDGEKAPS
VviHMT2  148 PDMN-LDKLKDFHRRRLQVLVR-SCPDLLAFETIPNKLEAQAC----------VELLEEENVQIPSWICFSSVDGENAPS
TcaHMT1  150 PEVN-LDKLKDFHRQRLQVLVK-AGPDLLAFETIPNKLEAQAC----------VELLEEENIQIPSWICFSSVDGENAPS
GmaHMT4  146 PDVN-LKKLKDFHRRRLQVLVE-AGPDLLAFETIPNKLEAQAC----------VELLEEESVKIPSWICFTTVDGENAPS
PvuHMT1  149 PDVD-LKKLKDFHRRRLQVLVE-AGPDLLAFETIPNKLEAQAC----------VELLEEESVNIPSWICFTTVDGENAPS
MtrHMT2  149 PDVS-LVKLKDFHRRRLQVLVE-AGPDLLAFETIPNKLEAQAC----------VELLEEINVQIPSWICFTSVDGENAPS
MdoHMT1  151 PRVD-VDKLKDFHRRRLQVLVE-AGPDLLAFETIPNKLEAQAC----------VELLEEQNVQIPSWICFSSVDGENAPS
AcoHMT3  154 PNVN-LDKLKDFHRRRVQVLVE-AGADVLAFETIPNKLEAQAC----------AELLDEEHIQIPSWICFSSVDGEHASS
ZmaHMT4  144 ADIT-AEKLKDFHRRRLQVLAS-AGPDLIAFEAIPNQMEAQAL----------VELLEEEKVQIPSWICFSSVDGKNLCS
SbiHMT1  144 ADIT-AEKLKDFHRRRLQVLAS-AGPDLIAFEAIPNKMEAQAL----------VELLEEEKVQVPSWICFSSVDGKNLCS
OsaHMT3  145 EDIT-AEKLKDFHRRRLQVLAS-AGPDLIAFEAIPNKMEAQAL----------VELLEEENIQVPSWICFSSVDGKNLCS
BdiHMT1  145 DNIM-AEKLKDFHRRRLQVLAS-AGPDLIAFEAIPNKMEAQASFRSNGRVQALVELLEEEDIQVPSWICFSSVDGKHLCS
MacHMT1  145 PDMN-LEKLKEFHRRRLQVLVD-AGPDLLAFETIPNKLEAQAL----------AELLDEENIQIPSWICFSSVDGEHMSS
MpoHMT   164 PEMT-LEKLKDFHRERLLILAG-AGPDILALETIPSFLEAKAL----------IEVLEEEDINVPAWMSYISKDGRNVVR
SmoHMT2  152 PGMT-LKKLKDFHRRRLQILSS-CGPDLLAIETIPSKLEAQAF----------IELLGEEDIDVPAWIAFSSKDGKNVVS
PpaHMT1  161 PSMT-VDKLKDFHRRRLVVLAD-AGPDLLAIETIPCKLETQAL----------VELLHEEDLRIPAWISFNSKDGVNVVS
PpaHMT3  167 PSMT-VDKLKDFHRRRLMVLAD-AGPDLIALETIPCKLETQAL----------VELLAEENLRVPAWISFNSKDGTNVVS
PpaHMT2  167 SFVT-VEKLKNFHRRRLLVLAD-AGPDLLAFETIPCKLEIQAL----------VELLDEEKIRIPAWVALNSKDGVNVVN
SfaHMT1  167 PNMT-IEKLKDFHRGRLLVLAN-AGADLLALETIPCKLETQAL----------IELLTDEDVPIPAWISFNSKDGTNVVK
SfaHMT2  164 PDMT-KTKLKDFHRRRLLVLAD-AGADLLAFETIPCKLEAQAF----------VEILEEEDIQIPSWLVFNSKDGLNVVN
SmoHMT1  151 PEMMNVAKLKGFHRRRLQILAS-SGADLLAIETIPCQVEAQAL----------VELLEEEDIQIPSWISFNSKDGANVVS
VcaHMT   158 DSMT-LQQLANFHRDRLEPVRHRTEIDLLAFETVPCLREAEAI----------LELLRQERYGKPAWISFSCRDAVHTSH
CreHMT   218 DSVS-TDALIEFHRARLDPVRSRHEIDLIAFETVPCLKEAEAI----------VELLRRERYCKPAWISFSCKDDAHTCH
EcoHMT   141 --CS-VEAFQAFHRPRVEALLD-AGADLLACETLPNFSEIEAL----------AELLTAYPRAR-AWFSFTLRDSEHLSD
PabHMT1   97 SDMT-LDKLKDFHRRRLQVLVE-AGPDLLAFETIPNKLEAQAC----------IELLEEEDVQVPSWLCFSAIDGIHVSS
PabHMT2  121 PGMT-VHTLKDFHRGRVQVLAD-SGADLLAFETIPNKLEAQAY----------IELLEENDIQIPAWFSFNSKDGVNVVS
PtaHMT1  160 PGMT-VQALKDFHRRRVQVLAE-SGADLLAFETIPNKLEAQAY----------IELLEENNIQIPAWFSFNSKDGVNVVG
PtaHMT2  156 SDVT-LDKLKDFHRRRLQVLVE-AGPDLLAFETIPNKLEAQAC----------VELLEEEDVQVPSWLCFSAIDGIHVSS
AtrHMT1  155 VDVT-LDTLKDFHRRRVQVLAE-SGADLIAFETIPNKLEARAY----------VQLLEENDINIPAWFTFNSKDGTNVVS
AtrHMT2  145 PDVS-LEKLKDFHRRRLQVLVE-AGPDMLAFETIPNKLETQAC----------VELLHEENIKIPSMICFSSVDGEHAAS
SpoHMT1  148 PDVG-LAKLKDFHRRRLQVLVG-AGPDILAFETIPNKIEAQAL----------VELLDEEEIKIPSLICFSSVDGERLSS
SpoHMT2  160 DAIT-LKWLKDFHRRRVQILAE-SGADLLAFETIPNKLECQAY----------AELLEENDIRIPAWFAFNSKDGINVVS
ZomHMT   154 EAID-LEALKDFHRERFLILAD-SGADLIAFETIPNKLECQAY----------AELLKENDIKIPAWFAFNSKDGVHVVS
                       

            


GmaHMT1  222 GDSLM-ECGSIAESCN----KVVAVGINCTPPRFIHGLIVLLKK------VTTK--------------------------
GmaHMT2  201 GDSLM-ECGSIAESGN----KVVAVGIYCTPPRFIHGLIVLLKR------VTTK--------------------------
PvuHMT3  218 GDSLA-ECGSIAESCK----NVVAVGINCTPPRFIHDLIVLLKK------VTTK--------------------------
MtrHMT1  224 GDSIV-ECGSIAESCN----KVIAVGINCTPPRFIHGLILLLKK------VTTK--------------------------
AbiHMT   222 GDSIE-ECGSIAESCD----KVVAVGINCTPPRFIHDLILLLKK------VTAK--------------------------
SlyHMT2  230 GDSLS-ECAAIGESCE----KVLAVGINCTPPRFILDLILSIKQ------VTTK--------------------------
VviHMT1  211 GDSLL-ECVSIAESCK----KVVSVGINCTPPRFIHGLILSIKK------VTTK--------------------------
MdoHMT3  229 GDSLL-ECATVAESCK----KVVAVGINCTPPRFIHGLLTLITQ------VATK--------------------------
MdoHMT4  218 GDSLL-ECASVAESCK----KVVAVGINCTPPRFIHGLLTLITQ------VATK--------------------------
TcaHMT2  229 GDSLL-ECASIAESCK----QVVAVGINCTPPRFIHELILAIKK------VTTK--------------------------
BraHMT4  231 GDSIK-ECVAIAEACE----KVVAVGINCTSPRFIEGLVLEIAK------VTSK--------------------------
BolHMT1  231 GDSIK-ECVAIAEACE----TVVAVGINCTSPRFIEGLVLEIAK------VTSK--------------------------
BraHMT1  224 GDSIK-ECISIAETCE----KVVAVGINCTPPRFIEGLVLEIAK------VTSK--------------------------
BolHMT2  225 GDSIK-ECIAIAEACE----KVVAVGINCTPPRFIEGLVLEIAK------VTSK--------------------------
AthHMT2  222 GDSIK-ECISIAENCE----KVVAVGINCTPPRFIEGLVLEIEK------VTSK--------------------------
AcoHMT1  229 GDSVI-ECASIAESCE----KAIAVGINCTPPRFIRGLILSIQK------VTTK--------------------------
GmaHMT3  227 GDSIF-ECASIADSCR----QVVAVGVNCTAPRFIHGLISFIKKQ-----ATSK--------------------------
PvuHMT2  228 GDSIF-ECASIADSCR----QVAAVGVNCTAPRFIHGLISSIKK------ATSK--------------------------
MtrHMT3  217 GDSIL-ECASIADSCP----QVVAVGVNCTAPRFIHGLISSIKK------VTSK--------------------------
MdoHMT2  180 GDSML-ECASIADSCK----NVVAVGINCTPPRFIYGLVSSIRN------VTNK--------------------------
TcaHMT3  227 GDSIC-DCASIADSCK----QVAAVGINCTPPRFIHGLILSIRK------VTNK--------------------------
BraHMT2  229 GESII-ECAEVADSCK----KLVAIGINCTAPRYISDLVISLRQ------VTHK--------------------------
BolHMT4  229 GESII-ECAEVADSCK----KVVAIGINCTAPRYISDLIISLRQ------VTHK--------------------------
AthHMT3  228 GDSVV-ECAKVADSCK----NVVAIGINCTAPRYIHALIISLRQ------MTRK--------------------------
SlyHMT1  226 GDSIA-ECASIVDSCK----QVVGIGINCTSPRYIQGLIQSIRK------VTSK--------------------------
VviHMT3  220 GDSLI-ECASIADSCK----QVVAVGINCTPPRFIHGLILLIQK------VTTK--------------------------
AcoHMT2  233 GDSLL-DCASVADKCN----KVLAVGINCTPPRFIHGLIQLIKK------VTSK--------------------------
ZmaHMT1  221 GDSLI-ECTTIADKCA----KVGAVGINCTPPRFIHGLILSIRK------VTDK--------------------------
ZmaHMT3  221 GDSLI-ECATIADKCA----KVGAVGINCTPPRFIHGLILSIRK------VTDK--------------------------
SbiHMT2  221 GDSVI-ECTTIADKCA----KVGAVGINCTPPRFIHGLILSIRK------VTDK--------------------------
OsaHMT4  225 GDSLI-ECATIANGCS----KVGAVGINCTPPRFIHGLILSIRK------VTDK--------------------------
BdiHMT2  228 GDSLI-ECATIANACA----KVGAVGINCTPPRFIHGLILSIRK------VTDK--------------------------
OsaHMT1  227 GDSLI-ECASIADSCK----EVAAVGINCTPPRFIHELVLSIRK------VTSK--------------------------
MacHMT4  218 GDSLM-DCASIADSCD----NVVAIGFNCTSPRYIHSLILSIRK------LTEK--------------------------
MacHMT2  224 GDSLI-ECASLADSCK----KVVAIGINCTPPRFIQGLILSIRK------VTKK--------------------------
MacHMT3  224 GDSLT-ECVSIADSCK----KVVAVGINCTPPRFIHGLILSIKK------VTKK--------------------------
ZmaHMT2  222 GDPIN-ECAAVADSCP----RVAAVGVNCTAPRFIHGLILSIKK------VTSK--------------------------
SbiHMT3  207 GDPIT-ECAAVADSCQ----RVAAVGVNCTSPRLIHGLILSIKK------VTSK--------------------------
BdiHMT3  230 GDPIT-ECAAVADSCR----RVASVGINCTAPGLIHGLILSIRK------VTSK--------------------------
OsaHMT2  200 GDPIT-ECAAVADACA----RVGAVGVNCTAPRLVHGLILSIRK------VTSK--------------------------
BraHMT3  218 GESFE-ECLETLNKSN----NICAVGINCAPPQFMDNLIRKFSK------LTKK--------------------------
BolHMT3  218 GESFQ-ECLETLNKSN----NICAVGINCAPPQFMDNLIRKFSK------LTQK--------------------------
AthHMT1  218 GESFE-ECLEPLNKSN----NIYAVGINCAPPQFIENLIRKFAK------LTKK--------------------------
VviHMT2  216 GESFK-ECLDIINKSK----KVNAVGINCAPPHFLESLICKFKE------LTEK--------------------------
TcaHMT1  218 GESFK-ECLDILNKSV----KVNAVGINCAPPHFIESLVCKFKE------MTSK--------------------------
GmaHMT4  214 GESFK-DCLEALNKSN----KVDAVGINCAPPHLMENLICKFKQ------LTKK--------------------------
PvuHMT1  217 GESFK-DCLEAINKSI----KVDAVGINCAPPHFIESLICTFKQ------LTKK--------------------------
MtrHMT2  217 GESFQ-YCLEVINKSN----KVEAVGINCAPPHFMESLIPKFKQ------LTNK--------------------------
MdoHMT1  219 GEGFT-ECLEVINKSN----KIHAVGINCTPPHLIQSLICKFKEAVSVLQLTSK--------------------------
AcoHMT3  222 GESFR-ECLDILNRSD----KVNAVGINCTPPHFIENLISNFRE------LTDK--------------------------
ZmaHMT4  212 GESFA-DCLKILNASE----KVAVVGVNCTPPQFIEGIICEFRK------QTKK--------------------------
SbiHMT1  212 GESFA-DCLKILDTSD----KVAVVGVNCTPPQFIEGIICEFKK------QTKK--------------------------
OsaHMT3  213 GESFA-ECLQFLNASD----KVTIVGVNCTPPQFIEGIIRELKK------QTKK--------------------------
BdiHMT1  223 GESFG-DCLQILNASE----KVAIVGVNCTPPQFIEGIIREFKK------QTGK--------------------------
MacHMT1  213 GEDFK-ECLDILNRNE----KVNIVGINCASPQFIENLILKFKK------LTNK--------------------------
MpoHMT   232 GDSIQ-SCAELVERSE----KIVAFGINCTPPSLCEELISEARK------ITSK--------------------------
SmoHMT2  220 GDNFS-ESIAMLDKCD----KVVAVGINCCPPHFVEGLIHEARK------ATSK--------------------------
PpaHMT1  229 GDSFS-DCVALVDKCP----EVAAVGINCTPPRFILDLIHAARK------VTNK--------------------------
PpaHMT3  235 GDSLS-DCVALADKCT----QVRAVGINCTPPRFILDLIQAVRK------VTNK--------------------------
PpaHMT2  235 GDSLT-DCVGLLDNCT----KVVAVGINCTPPRFILDLIRVARK------VTSK--------------------------
SfaHMT1  235 GDSFD-ECVGLADKCK----KIVAVGINCTPPRFIHDLICTVRK------ATLK--------------------------
SfaHMT2  232 GDSFD-ECIALAANSK----KVVAVGINCTPPRFIDGMIRAARK------VTSK--------------------------
SmoHMT1  220 GDPLS-ECVALAAKSA----KVAAVGINCTPPRFIHGLVSTARK------VTDK--------------------------
VcaHMT   227 GERFAEQCVPLLAAAAAEGLEVVATGVNCTAPRHVRALLTAARAQLNAAAAATTAATTAGTTAATAAATATPIAFEKIVE
CreHMT   287 GEDFGAQCVPLLAAAAAEGL-VAAAGVNCTPPRHVPALLAAARQQLQQLEVS-------------AAAPPPP--------
EcoHMT   206 GTPLR-DVVALLAGYP----QVVALGINCIALENT----TAALQHLHGLTVLP---------------------------
PabHMT1  165 GETFR-DCLDAVEKSE----KVVGIGINCSPPNFIEDLIQIARK------ATEK--------------------------
PabHMT2  189 GDSFT-ECAALADSCT----NVVAVGINCTPPRFIHGLILSIQK------VTS---------------------------
PtaHMT1  228 GDSFT-ECAALADSCP----NVVAVGINCTPPRFIHGLILTIQK------VTAK--------------------------
PtaHMT2  224 GETFR-DCLDAVEKSE----KVVGIGINCSPPNFIEDLIRIARK------ATEK--------------------------
AtrHMT1  223 GDSLL-ECASIADSCK----KVVAVGINCTPPRYIHDLILFIKK------VTTK--------------------------
AtrHMT2  213 GESFH-ECLDLLNKCD----KVCAVGINCTPPHFIENLIHMFLK------LSRK--------------------------
SpoHMT1  216 GESFA-DCLEIMNKSE----KVAAVGLNCTPPQFMEHLICLFKK------HTDK--------------------------
SpoHMT2  228 GDSIP-ECISIAEQCT----KVVAVGINCTPPRFIHGLLLSIQK------VTTK--------------------------
ZomHMT   222 GDSFV-ECISIAESCK----QVVAVGINCTPPRFIHGLLMTIRK------VTTK--------------------------
                           

  
GmaHMT1  265 ------------------------------PIVIYPNSGETYDADLKEWVQ------------NTGVTDEDFISY-VNKW
GmaHMT2  244 ------------------------------PIVIYPNSGETYDADLKEWVQ------------NTGVTDEDFISY-VNKW
PvuHMT3  261 ------------------------------PIVIYPNSGETYDAELKEWVQ------------NTGETDENFISY-VTKW
MtrHMT1  267 ------------------------------PIAIYPNSGETYDGERKEWMQ------------NTGVTDKDFVSY-VSKW
AbiHMT   265 ------------------------------PIVIYPNSGETYDAIRKEWGQ------------NSGVTDEDFVSY-VDKW
SlyHMT2  273 ------------------------------PILIYPNSGESYDGIRKEWVS------------NTGVTDEDFVPY-VNKW
VviHMT1  254 ------------------------------PILIYPNSGESYDPEQKEWVQ------------KTGVSVEDFVSY-VNKW
MdoHMT3  272 ------------------------------PIIVYPNSGESYDPDRKMWVQ------------NTGVSDEDFVSY-VNKW
MdoHMT4  261 ------------------------------PIIVYPNSGESYDADRMMWVE------------VVAEQPQIQSEQ-YTGL
TcaHMT2  272 ------------------------------PIIIYPNSGERYDADQKEWVGDHALTNEPNAQENTGVSDEDFVSY-VSKW
BraHMT4  274 ------------------------------PILAYPNSGERYDADRKEWVE------------NAGVGDEDFVSY-VEKW
BolHMT1  274 ------------------------------PILAYPNSGERYDADRKEWVE------------NAGVGDEDFVSY-VEKW
BraHMT1  267 ------------------------------PILVYPNSGERYDPERKEWVE------------NTGVGNEDFVSY-VEKW
BolHMT2  268 ------------------------------PILVYPNSGERYDPERKEWVE------------NTGVGNEDFVSY-VEKW
AthHMT2  265 ------------------------------PILVYPNSGESYDADRKEWVE------------NTGVGDEDFVSY-VEKW
AcoHMT1  272 ------------------------------PILIYPNSGETYDPDRKQWVQ------------STGVSDEDFVSY-VSKW
GmaHMT3  271 ------------------------------PVLVYPNSGETYIAESNQWVK------------SSGAAEHDFVSY-IGKW
PvuHMT2  271 ------------------------------PVLVYPNSGETYIAESNQWVK------------SSGLAEDDFVPY-IGKW
MtrHMT3  260 ------------------------------PILVYPNSGETYNAENNTWVK------------SSGEAEEDFVPY-IGKW
MdoHMT2  223 ------------------------------PIVIYPNSGETYDGQTKQWVK------------STGVVNEEFADIAIDKW
TcaHMT3  270 ------------------------------PVVIYPNSGETYDAERKTWVK------------STGVADEDFVSY-AAKW
BraHMT2  272 ------------------------------PIIVYPNSGEVYDGLNKKWIK------------SEEESEEDFVSY-VSRW
BolHMT4  272 ------------------------------PIIVYPNSGEVYDGLNKKWIK------------SEEESEEDFVSY-VSRW
AthHMT3  271 ------------------------------PIVVYPNSGEVYDGLNKKWIK------------SEGESEEDFVSY-VSKW
SlyHMT1  269 ------------------------------PILVYPNNGETYDGVKKEWVA------------SRGVVEEDFVSY-VDKW
VviHMT3  263 ------------------------------PVVIYPNSGETYDGVRKEWVK------------SSGVQDGDFVSY-VSKW
AcoHMT2  276 ------------------------------PILVYPNSGEMYDPDRKLWVQ------------SAGVSDEDFVSH-VNKW
ZmaHMT1  264 ------------------------------PILIYPNSGERYDGEKKEWVE------------STGVSDGDFVSY-VNEW
ZmaHMT3  264 ------------------------------PILIYPNSGERYDGEKKEWVE------------STGVSDGDFVSY-VNEW
SbiHMT2  264 ------------------------------PILIYPNSGERYDGEKKEWVE------------STGVSDGDFVSY-VNEW
OsaHMT4  268 ------------------------------PILIYPNSGERYDAEKKEWVE------------STGVSDGDFVSY-VNEW
BdiHMT2  271 ------------------------------PILIYPNSGERYDAEKKEWVE------------STGVCDGDFVSY-VSEW
OsaHMT1  270 ------------------------------PILIYPNSGESYDPIRKEWVE------------CSGISNEDFVSY-VKKW
MacHMT4  261 ------------------------------LIVIYPNSGESYDPDKKEWVA------------SNGVSGEDFVSY-VSKW
MacHMT2  267 ------------------------------PILIYPNSGERYDAEKKEWVT------------STGVTDEDFVSY-VRVW
MacHMT3  267 ------------------------------PILIYPNSGERYDADKKEWVE------------STGVTDEDFVSY-VQVW
ZmaHMT2  265 ------------------------------PIVVYPNSGESYVAETNEWVD-----------SDGATGTDDFVSR-VGEW
SbiHMT3  250 ------------------------------PIVVYPNSGETYIADTNEWVD-----------SDGATGT-DFVSS-VGEW
BdiHMT3  273 ------------------------------AIVVYPNSGETYVAETKEWVD-----------SAGASGTTDFASC-VGKW
OsaHMT2  243 ------------------------------PVVVYPNSGETYVAETKEWVE-----------SEGGASETDFVSC-VGKW
BraHMT3  261 ------------------------------AIVVYPNSGEVWDGKAKKWLP------------SQCFGDAEFEMF-ATKW
BolHMT3  261 ------------------------------AIVVYPNSGEVWDGKAKKWLP------------SQCFGDAEFEMF-ATKW
AthHMT1  261 ------------------------------AIVVYPNSGEVWDGKAKQWLP------------SQCFGDDEFEMF-ATKW
VviHMT2  259 ------------------------------PIVVYPNSGEVWDGRAKRWLP------------SKCFGDDKFELY-ATKW
TcaHMT1  261 ------------------------------AIVVYPNSGEIWDGRAKKWLP------------SKCFGDDKFELF-AARW
GmaHMT4  257 ------------------------------AIIVYPNSGEVWDGKAKKWLP------------SKCFHDDEFGFN-ATRW
PvuHMT1  260 ------------------------------AIIVYPNSGGVWDSKAKKWQL------------PKVFNDDDFGFN-ATRW
MtrHMT2  260 ------------------------------AIVVYPNSGEVWDGIAKKWLP------------SKCFHDDDFGFY-ATRW
MdoHMT1  268 ------------------------------AIIVYPNSGEIWDGKAKRWLP------------AKCFDDENFECF-ATIW
AcoHMT3  265 ------------------------------PIVVYPNSGEVWDGKDKRWLP------------SKCFGHETFELY-ATRW
ZmaHMT4  255 ------------------------------AIAVYPNSGEVWDGRAKRWLP------------VECLGHKSFDAL-AKRW
SbiHMT1  255 ------------------------------AIAVYPNSGEVWDGRAKRWLP------------VECLGHKSFDAL-AKRW
OsaHMT3  256 ------------------------------AIAVYPNSGEIWDGRAKRWLP------------AQCFGHKSFDAL-AKRW
BdiHMT1  266 ------------------------------AIAVYPNSGEVWDGRAKRWLP------------AECFGRKSFDVM-ARRW
MacHMT1  256 ------------------------------LLAVYPNSGEVWDGRAKRWLQ------------PERVGEKSFEFL-AKRW
MpoHMT   275 ------------------------------PIVVYPNRGEDWNAEKKEWIP------------STGATDEDFINY-IPRW
SmoHMT2  263 ------------------------------TIVVYPNSGEQYDPKTKLWKV------------QERNCEKDFMAF-VKNW
PpaHMT1  272 ------------------------------PIVVYPNSGEHYDPVIKQWVE------------STGITDTDFVSY-VHEW
PpaHMT3  278 ------------------------------LIVVYPNSGEYYDPEIKQWVE------------STGVSDTDFVSY-VHEW
PpaHMT2  278 ------------------------------PIMVYPNSGEHYDAVIKQWVE------------CKGSTDTDFVSH-VQEW
SfaHMT1  278 ------------------------------PIVVYPNSGEQYDPSIKQWVE------------STGVSDTDFVSY-VSEW
SfaHMT2  275 ------------------------------PIVVYPNSGECYDGIKKEWIT------------CTGMGEKHFISH-VAEW
SmoHMT1  263 ------------------------------PIVVYPNSGETFDPDAKQWIP------------STGVSDVDFVSY-VGEW
VcaHMT   307 PHMQRELQEAAKQQQQQQQKQPQLRPPSQLLLVCYPNSGEEWDGEHRCWRH-----------LPDDIAEPECFAEAAAEC
CreHMT   345 ---------SPPADGMRQQQQHLACAPRRLLLLCYPNSGEQWDGAGRCWHS-----------APDDIAEPGRFAAAAADW
EcoHMT   250 -------------------------------LVVYPNSGEHYDAVSKTWHH-----------HGEHCAQ---LADYLPQW
PabHMT1  208 ------------------------------AIVVYPNSGEIWDGKAKQWLP------------SKCFCEEQFGIL-VKRW
PabHMT2  231 --------------------------------LDYVN----------------------------GTDVLFFCHF-VG--
PtaHMT1  271 ------------------------------PILVYPNSGETYDAERKQWVA------------SSGVSDIDFVSY-VQKW
PtaHMT2  267 ------------------------------AIVVYPNSGEIWDGKAKQWLP------------AKCSCEEQFEIL-VKRW
AtrHMT1  266 ------------------------------PIVIYPNSGETYDSVIKEWVE------------SAGVSDEDFVSH-VEKW
AtrHMT2  256 ------------------------------IIAVYPNSGEVWDGRTKKWLH------------STCFGDKEFGSY-AKRW
SpoHMT1  259 ------------------------------LIAVYPNSGEIWDGRAKKWLP------------SECFGDETFTVL-AKRW
SpoHMT2  271 ------------------------------PILIYPNSGETYDSDAKKWVE------------TSGESDEDFVSY-VTKW
ZomHMT   265 ------------------------------PILIYPNSDETYDPDVKEWVE------------STGVSDDDFVSY-VTKW
                                                                                              
GmaHMT1  302 CEL---GASLVGGCCRTTPDTIRKIYRTLSSSQSI------------------------------------
GmaHMT2  281 -----------------------------------------------------------------------
PvuHMT3  298 REL---GASLVGGCCRTTPATIRKIYSVLSSKESAILGKE-------------------------------
MtrHMT1  304 CEL---GACLVGGCCRTTPVTVRGIYNTLYSNQSAILSTQ-------------------------------
AbiHMT   302 CES---GASLVGGCCRTTPDTIRGIYKILS-----------------------------------------
SlyHMT2  310 CEA---GASLVGGCCRTTPNTIRAIYKSLSGKRTV------------------------------------
VviHMT1  291 CEV---GASLVGGCCRTTPNTIRAIYRTLSNNRSATASLKS------------------------------
MdoHMT3  309 CEA---GASLVGGCCRTTPNTIRAIYTTLPNRSTSPPEQ--------------------------------
MdoHMT4  298 FPI---GQHLH-LICNHIGFAKRAVEQDYEKACTEKIASCL------------------------------
TcaHMT2  321 CEI---GASLVGGCCRTTPDTIRAIYRTLSPRSPDPPMQ--------------------------------
BraHMT4  311 MDA---GVSLLGGCCRTTPATIRAIHKRLVTRRSLFSFS--------------------------------
BolHMT1  311 MDA---GVSLLGGCCRTTPATIRAIHKRLVTRRSLFSSS--------------------------------
BraHMT1  304 MDA---GVSLLGGCCRTTPTTIRAIHKRLVSRRSLFSSSSSSSHH--------------------------
BolHMT2  305 MDA---GVSLLGGCCRTTPTTIRAIHKRLVSRRSLFSSSSSSSHH--------------------------
AthHMT2  302 MDA---GVSLLGGCCRTTPTTIRAIHKRLVNRRSL------------------------------------
AcoHMT1  309 REY---GASLIGGCCRTTPNTIKAIYKTLKSPADSPSQL--------------------------------
GmaHMT3  308 RDA---GASLFGGCCRTTPNTIRGIAEATYGKLKDKCI---------------------------------
PvuHMT2  308 RDA---GASLFGGCCRTTPNTIRGIAEAIYGKVQDKCI---------------------------------
MtrHMT3  297 RYA---GATLFGGCCRTTPKTIRGITEALYGKPHGKCI---------------------------------
MdoHMT2  261 CEA---GASLFGGCCRTTPNTIRAISKTLSNKSSSTVNDDA------------------------------
TcaHMT3  307 RDI---GASLFGGCCRTTPNTIRAISRILFDKSSLTLSKADM-----------------------------
BraHMT2  309 REE---GASLFGGCCRTTPNTIKAITNVLSCESSAPSKLKFW-----------------------------
BolHMT4  309 REE---GASLFGGCCRTTPNTIKAIAKVLSCESSVPSKLKFW-----------------------------
AthHMT3  308 RDA---GASLFGGCCRTTPNTIRAIAKVLSDEPSAASKPKFGQ----------------------------
SlyHMT1  306 CDA---GASLVGGCCRTTPNTIRAISKVLSRRSQSV-----------------------------------
VviHMT3  300 REA---GASLFGGCCRTSPHTIRAIS------MTLSSYLQ-------------------------------
AcoHMT2  313 REV---GASLFGGCCRTTPNTIRAISKILSKGSGAPFQQKESY----------------------------
ZmaHMT1  301 CKD---GAVLIGGCCRTTPNTIRAIHRTLNKSPNKQQLPAVE-----------------------------
ZmaHMT3  301 CKD---GAALIGGCCRTTPNTIRAIHRTLNQGCHKHQLPVA------------------------------
SbiHMT2  301 CKD---GAALIGGCCRTTPNTIRAIQRTLNQGFN-------------------------------------
OsaHMT4  305 CKD---GAVLIGGCCRTTPNTIKAISRSLNQRHSSLHLPVA------------------------------
BdiHMT2  308 CKD---GAALIGGCCRTTPNTIRAINRSLNQ-----CLPAP------------------------------
OsaHMT1  307 HEA---GASLIGGCCRTSPDTIRGISKALHGV---------------------------------------
MacHMT4  298 HEA---GASLIGGCCRTTPDTIRAIAKALRKDFYLLHNDTERNILKDSFLIDSA-----------------
MacHMT2  304 CEA---GACLIGGCCRTTPNTIRGISKVLQK----------------------------------------
MacHMT3  304 HEA---GACLIGGCCRTTPNTIRGISLALQKEHAIPRESVP------------------------------
ZmaHMT2  303 RRA---GAALIGGCCRTSPATVRAIARAVREA--------EYDDIPAVAVL--------------------
SbiHMT3  287 RRA---GAALIGGCCRTSPATVRAIARALREAD-----ADEYDDMPAVAVL--------------------
BdiHMT3  311 REA---GASVVGGCCRTSPATVGAIARALREADAADVFYRPKPCSLVFSQKS-------------------
OsaHMT2  281 RQA---GAALVGGCCRTSPATVRAISWALRESDDAVGGDGDRDDFPAVAVL--------------------
BraHMT3  298 RDL---GAKLIGGCCRTTPSTIKAICRDLKRR---------------------------------------
BolHMT3  298 RDL---GAKLIGGCCRTTPSTIKAISRDLKRR---------------------------------------
AthHMT1  298 RDL---GAKLIGGCCRTTPSTINAISRDLKRR---------------------------------------
VviHMT2  296 RDL---GAKLIGGCCRTTPSTIRAISKVLKEMS--------------------------------------
TcaHMT1  298 REL---GAKLIGGCCRTTPATVGAISKALKERS--------------------------------------
GmaHMT4  294 RDL---GAKIIGGCCRTTPSTIQIISNALREKS--------------------------------------
PvuHMT1  297 RDL---GAKIIGGCCRTTPSTIQILSNALREKS--------------------------------------
MtrHMT2  297 REL---GAKIIGGCCRTTPSTIQIISNALRENI--------------------------------------
MdoHMT1  305 RDS---GAKLIGGCCRTTPSTVQAISKVLKGQSQ-------------------------------------
AcoHMT3  302 RDA---GAKLIGGCCRTTPSTIQAISNVLQQRS--------------------------------------
ZmaHMT4  292 QEA---GASLIGGCCRTTPSTIRAVSKILKGRTGH------------------------------------
SbiHMT1  292 QEA---GASLIGGCCRTTPSTIRAVSKILKGKTGH------------------------------------
OsaHMT3  293 QEA---GASLVGGCCRTTPSTIRAVSKVLKGKT----SYSATQI---------------------------
BdiHMT1  303 QEA---GASLIGGCCRTTPSTIRAVSKALKGRDRALISLESSESV--------------------------
MacHMT1  293 HEC---GASLIGGCCRTTPCTIRSISKLLKNMS--------------------------------------
MpoHMT   312 KKA---GANLFGGCCQTTPATVRAIAKVLHPHPVSSN----------------------------------
SmoHMT2  300 KRA---GANVIGGCCRTTPDTVRGICSAIF-----------------------------------------
PpaHMT1  309 RKA---GAQLIGGCCRTTPNTIGAIYKALHEHPHVHVTN--------------------------------
PpaHMT3  315 RNA---GAQLIGGCCRTTPNTIEAISKALREHTHAHVTN--------------------------------
PpaHMT2  315 RKA---GAQLIGGCCRTTPNTIRAISRVLYEHTQVYAAK--------------------------------
SfaHMT1  315 RDA---GAQLIGGCCRTTPTTIQAISNVLHENLHLHTTM--------------------------------
SfaHMT2  312 CES---GANLIGGCCRTTPITIKAISKVLHDQALPTSSPSQVPTLQKSEKSCSDQTLRLNFVPLSPSPELH
SmoHMT1  300 KKA---GASLIGGCCRTTPATIRAIKKSLQK----------------------------------------
VcaHMT   376 VYG-DPRVSLMGGCCRTGPEHIRALRRWLQAQQHCQQ----------------------------------
CreHMT   405 VGSRAMAVSLVGGCCRTGPAHIAALRRRLVAPPEDRAAAAAAAPALQ------------------------
EcoHMT   285 QAA---GARLIGGCCRTTPADIAALKARS------------------------------------------
PabHMT1  245 REA---GANLIGGCCRTTPTTILAISKALKDTL--------------------------------------
PabHMT2  248 ---------LVR-----------------NEKNYIIHA---------------------------------
PtaHMT1  308 QEI---GASLIGGCCRTTPNTIKAISRAMNRKSLILPSQHA------------------------------
PtaHMT2  304 REA---GANLIGGCCRTTPTTIQSISKALKDTL--------------------------------------
AtrHMT1  303 REA---GASFIGGCCRTTPLTIKAISRALNKRSTNSSKLTCKQDLEELFN---------------------
AtrHMT2  293 QEA---GAKLIGGCCRTTPSTVAAISNALRKQS--------------------------------------
SpoHMT1  296 REC---GASLVGGCCRTTPSTIRALSAALKSGPPSS-----------------------------------
SpoHMT2  308 CED---GARLIGGCCRTTPNTIRAISRALGNPLILLL----------------------------------
ZomHMT   302 HEA---GASLIGGCCRTTPNTIRAIYKAFN-----------------------------------------
                        


The sites were marked with stars were Tyr94, Thr274, Cys350, Cys495, and Cys496, respectively.
