# Supplementary material for: Molecular Evolution and Expression Divergence of HMT Gene Family in Plants
Source: Int J Mol Sci. 2018 Apr 20;19(4):1248. doi: 10.3390/ijms19041248 (PMC5979542; doi:10.3390/ijms19041248)
Supplement: Supplementary file 1 [file ijms-19-01248-s001.zip › Additional file 3.pptx]

## Slide 1
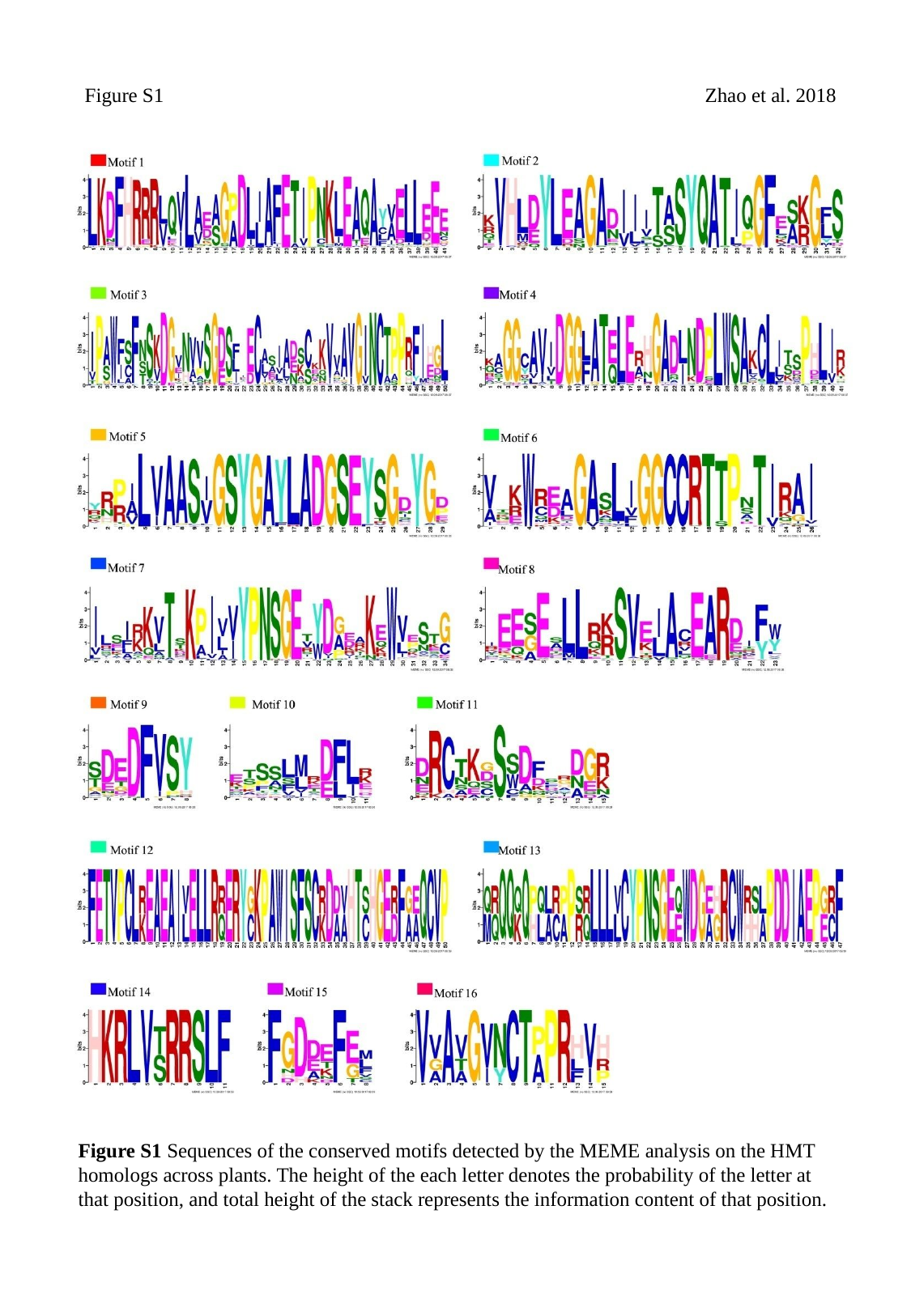

Figure S1 Zhao et al. 2018
Figure S1 Sequences of the conserved motifs detected by the MEME analysis on the HMT homologs across plants. The height of the each letter denotes the probability of the letter at
that position, and total height of the stack represents the information content of that position.

## Slide 2
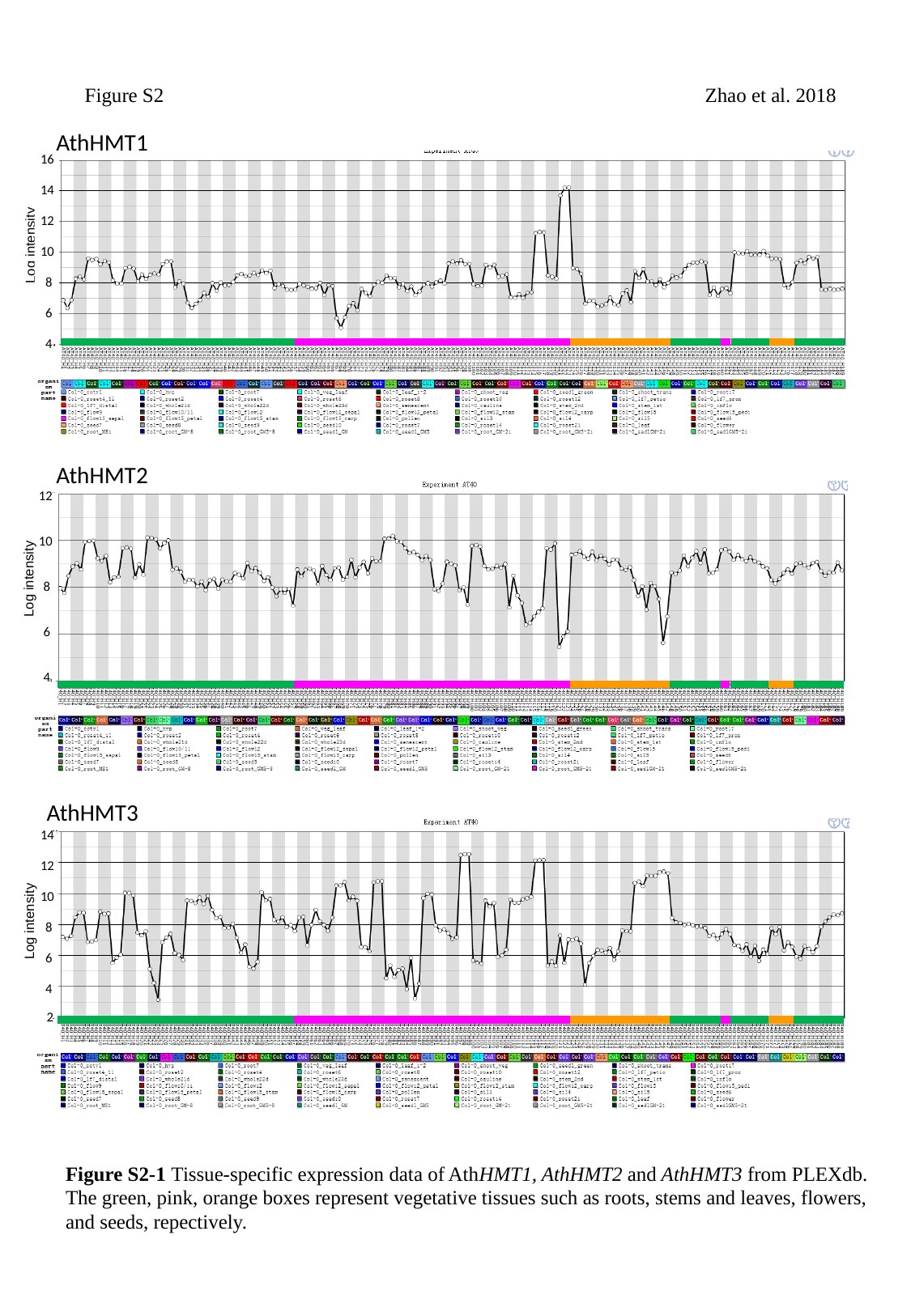

Figure S2 Zhao et al. 2018
AthHMT1
16
14
12
10
8
6
4
Log intensity
AthHMT2
12
10
8
6
4
Log intensity
AthHMT3
14
12
10
8
6
4
2
Log intensity
Figure S2-1 Tissue-specific expression data of AthHMT1, AthHMT2 and AthHMT3 from PLEXdb. The green, pink, orange boxes represent vegetative tissues such as roots, stems and leaves, flowers, and seeds, repectively.

## Slide 3
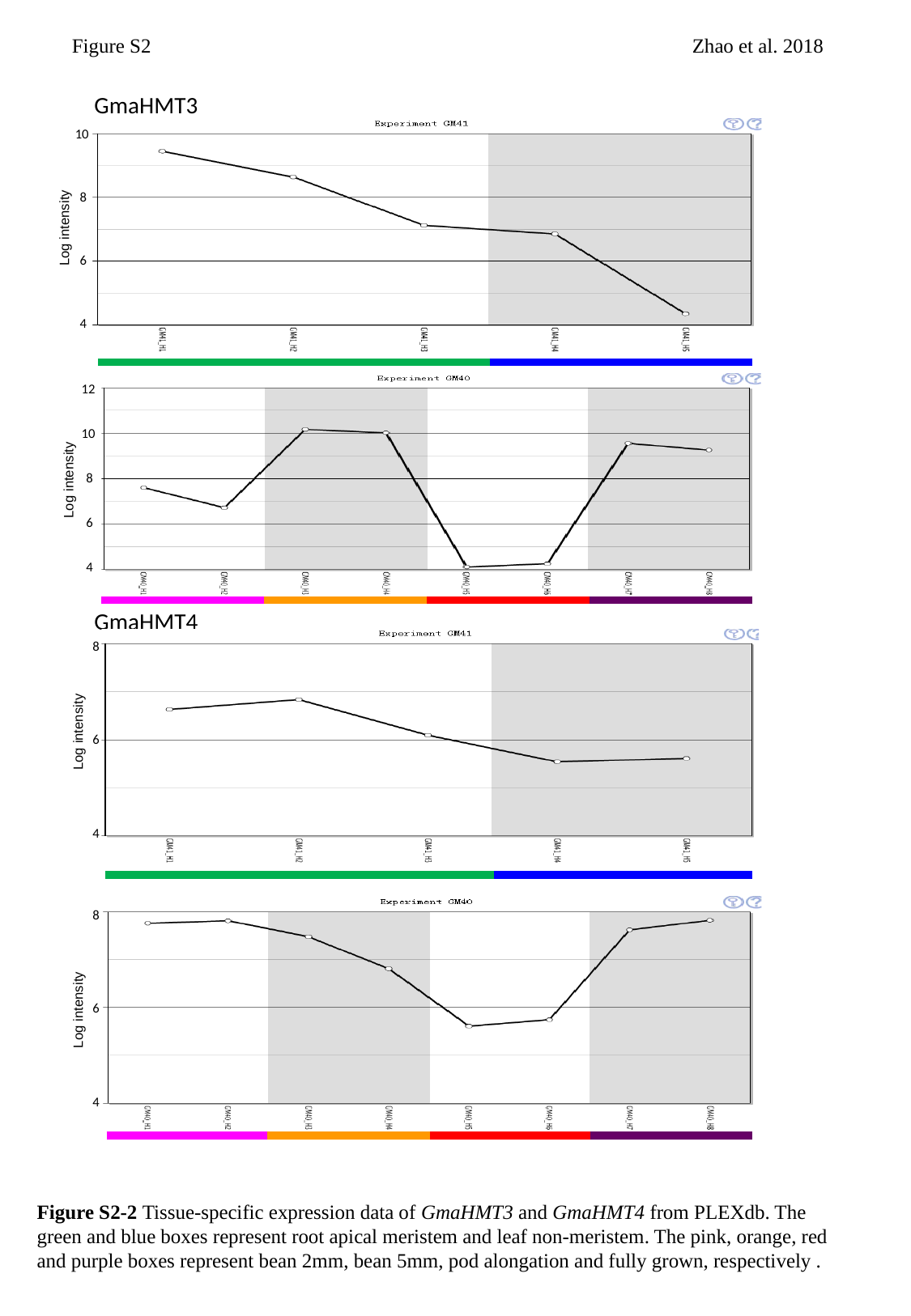

Figure S2 Zhao et al. 2018
GmaHMT3
10
8
Log intensity
6
4
12
10
Log intensity
8
6
4
GmaHMT4
8
Log intensity
6
4
8
Log intensity
6
4
Figure S2-2 Tissue-specific expression data of GmaHMT3 and GmaHMT4 from PLEXdb. The green and blue boxes represent root apical meristem and leaf non-meristem. The pink, orange, red and purple boxes represent bean 2mm, bean 5mm, pod alongation and fully grown, respectively .

## Slide 4
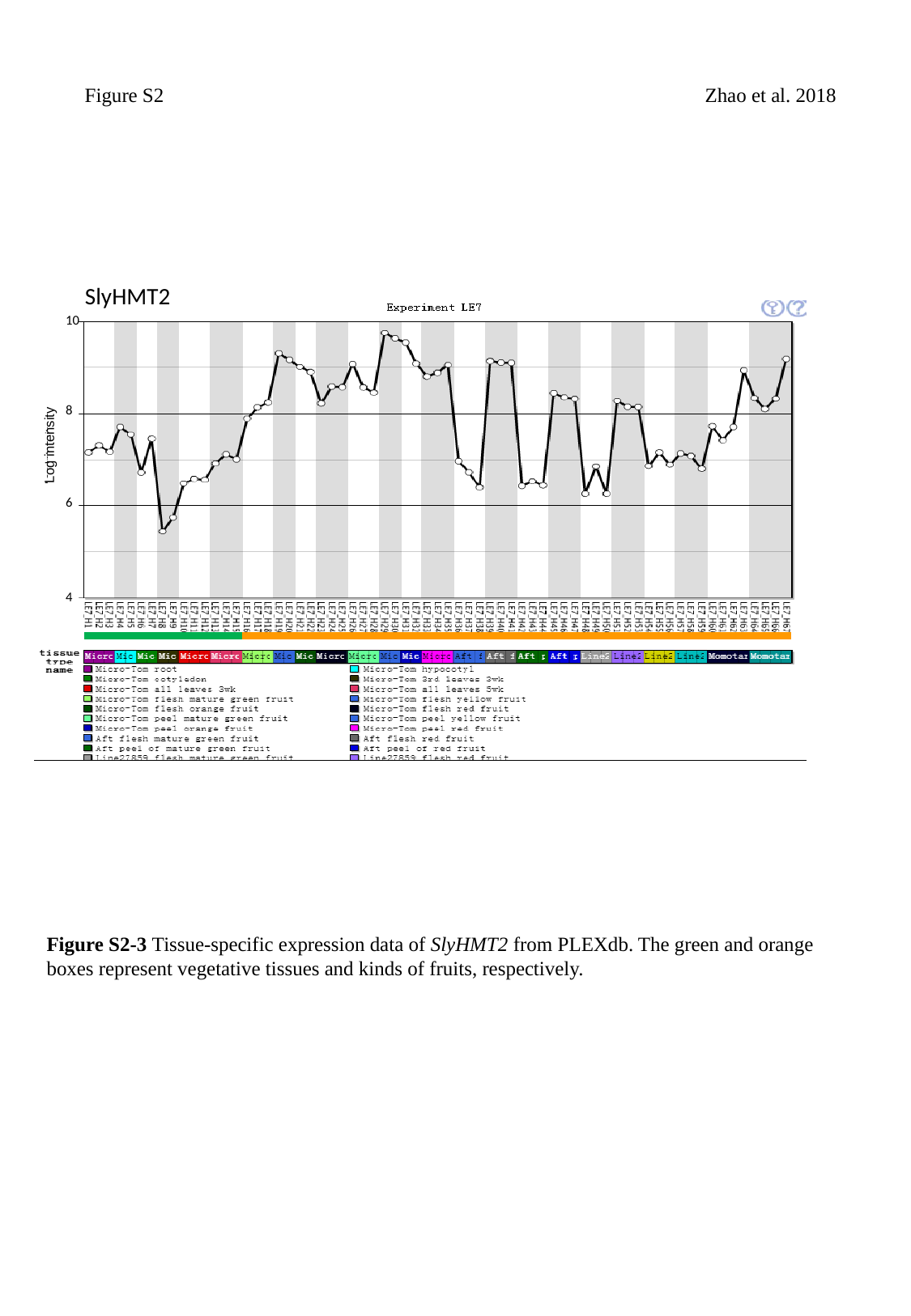

Figure S2 Zhao et al. 2018
SlyHMT2
10
8
6
4
Log intensity
Figure S2-3 Tissue-specific expression data of SlyHMT2 from PLEXdb. The green and orange boxes represent vegetative tissues and kinds of fruits, respectively.

## Slide 5
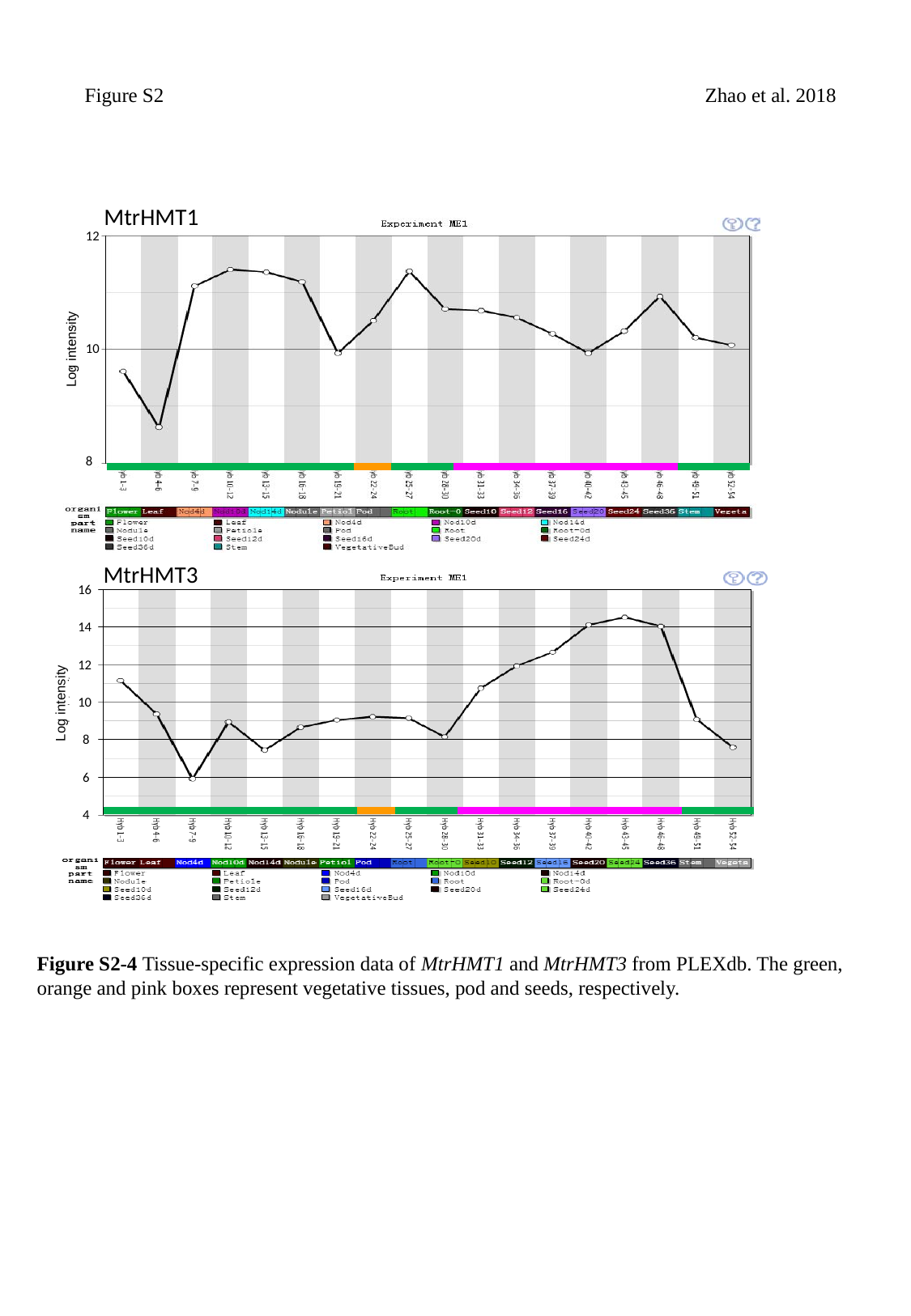

Figure S2 Zhao et al. 2018
MtrHMT1
12
10
8
Log intensity
MtrHMT3
16
14
12
10
8
6
4
Log intensity
Figure S2-4 Tissue-specific expression data of MtrHMT1 and MtrHMT3 from PLEXdb. The green, orange and pink boxes represent vegetative tissues, pod and seeds, respectively.

## Slide 6
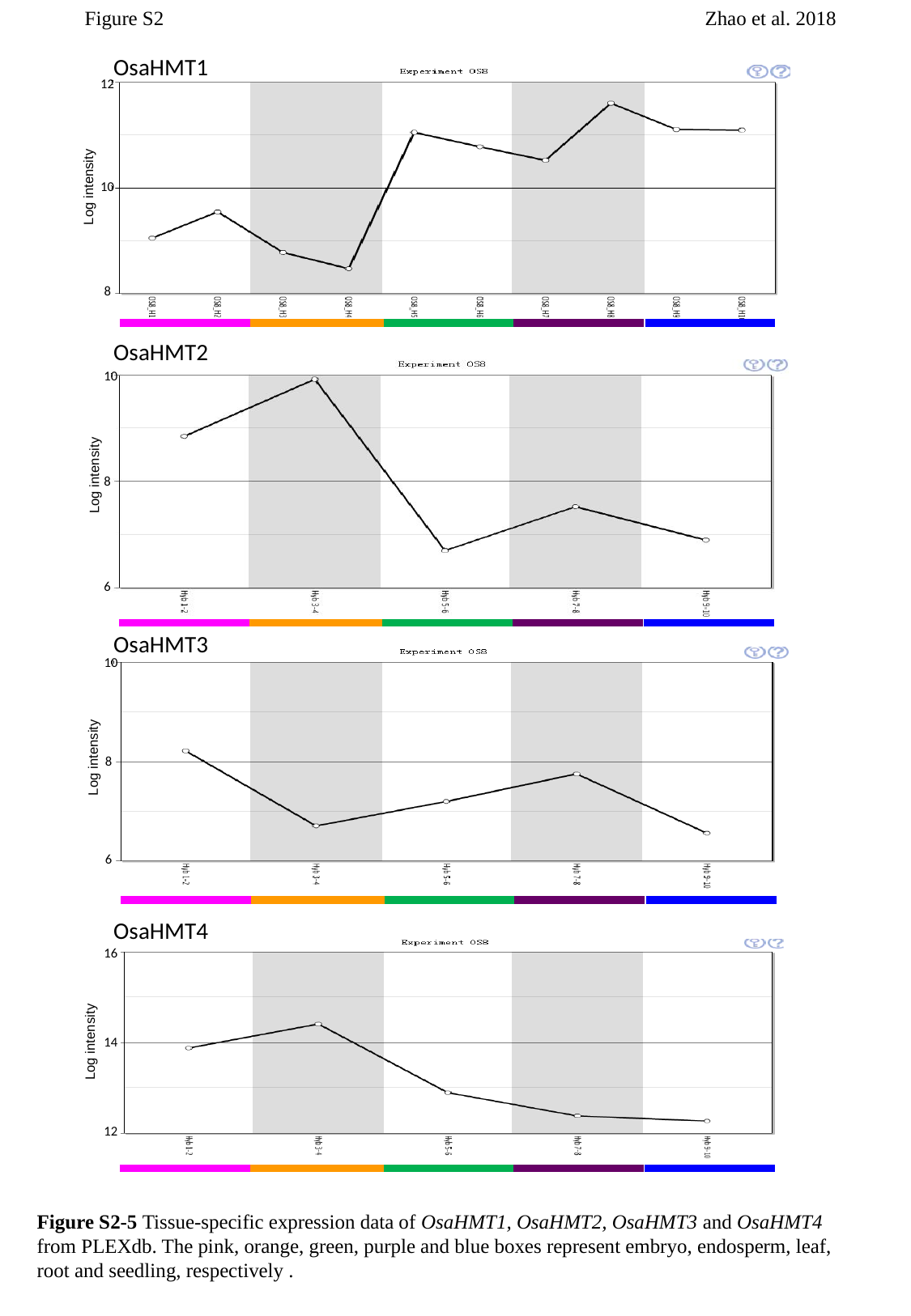

Figure S2 Zhao et al. 2018
OsaHMT1
12
Log intensity
10
8
OsaHMT2
10
Log intensity
8
6
OsaHMT3
10
Log intensity
8
6
OsaHMT4
16
Log intensity
14
12
Figure S2-5 Tissue-specific expression data of OsaHMT1, OsaHMT2, OsaHMT3 and OsaHMT4 from PLEXdb. The pink, orange, green, purple and blue boxes represent embryo, endosperm, leaf, root and seedling, respectively .
